# Episodic live imaging of cone photoreceptor maturation in GNAT2-EGFP retinal organoids

**DOI:** 10.1242/dmm.050193

**Published:** 2023-11-21

**Authors:** Jinlun Bai, David S. Koos, Kayla Stepanian, Zachary Fouladian, Dominic W. H. Shayler, Jennifer G. Aparicio, Scott E. Fraser, Rex A. Moats, David Cobrinik

**Affiliations:** ^1^ The Vision Center, Department of Surgery, Children's Hospital Los Angeles, Los Angeles, CA 90027, USA; ^2^ The Saban Research Institute, Children's Hospital Los Angeles, Los Angeles, CA 90027, USA; ^3^ Development, Stem Cell, and Regenerative Medicine Program, Keck School of Medicine, University of Southern California, Los Angeles, CA 90033, USA; ^4^ Translational Biomedical Imaging Laboratory, Children's Hospital Los Angeles, Los Angeles, CA 90027, USA; ^5^ Department of Radiology, Children's Hospital Los Angeles, Los Angeles, CA 90027, USA; ^6^ Department of Biomedical Engineering, Viterbi School of Engineering, University of Southern California, Los Angeles, CA 90089, USA; ^7^ Translational Imaging Center, University of Southern California, Los Angeles, CA 90089, USA; ^8^ Department of Ophthalmology and Roski Eye Institute, Keck School of Medicine, University of Southern California, Los Angeles, CA 90033, USA; ^9^ Department of Biochemistry and Molecular Medicine, Keck School of Medicine, University of Southern California, Los Angeles, CA 90033, USA; ^10^ Norris Comprehensive Cancer Center, Keck School of Medicine, University of Southern California, Los Angeles, CA 90033, USA

**Keywords:** GNAT2, Cone photoreceptor, Fluorescent reporter, Retinal organoid, iPSC, CRISPR, Live imaging

## Abstract

Fluorescent reporter pluripotent stem cell-derived retinal organoids are powerful tools to investigate cell type-specific development and disease phenotypes. When combined with live imaging, they enable direct and repeated observation of cell behaviors within a developing retinal tissue. Here, we generated a human cone photoreceptor reporter line by CRISPR/Cas9 genome editing of WTC11-mTagRFPT-LMNB1 human induced pluripotent stem cells (iPSCs) by inserting enhanced green fluorescent protein (EGFP) coding sequences and a 2A self-cleaving peptide at the N-terminus of guanine nucleotide-binding protein subunit alpha transducin 2 (*GNAT2*). In retinal organoids generated from these iPSCs, the *GNAT2-EGFP* alleles robustly and exclusively labeled immature and mature cones. Episodic confocal live imaging of hydrogel immobilized retinal organoids allowed tracking of the morphological maturation of individual cones for >18 weeks and revealed inner segment accumulation of mitochondria and growth at 12.2 μm^3^ per day from day 126 to day 153. Immobilized GNAT2-EGFP cone reporter organoids provide a valuable tool for investigating human cone development and disease.

## Teaser

**Summary:** We generated a human cone photoreceptor fluorescent reporter line and retinal organoids, and captured individual cone maturation through episodic live imaging, demonstrating the utility of the organoids as cone development and disease models.

## INTRODUCTION

Cone photoreceptors are critical for color vision and are impaired in retinal diseases such as cone-rod dystrophy, retinitis pigmentosa, Leber congenital amaurosis and retinoblastoma (Mustafi et al., 2009; Xu et al., 2014). Modeling of human cone development and disease in animals is challenged by human-specific cone development features (Singh et al., 2018). Human pluripotent stem cell (hPSC)-derived retinal organoids (ROs) closely recapitulate human retinal development, with similar developmental timeline, cellular composition and laminated structures (Aparicio et al., 2017; Bell et al., 2020). Combined with CRISPR gene editing, ROs provide opportunities to build cone disease models for mechanistic studies and therapeutic screening and are a potential source for retinal cell or tissue transplantation (Gasparini et al., 2019).

hPSC-derived ROs with cell type-specific fluorescent reporters can be used to monitor a cell's normal and disease-related behaviors. Photoreceptor reporter lines have been generated by introducing a *CRX-*GFP cassette (Kaewkhaw et al., 2015), a *RCVRN-eGFP* cassette (Guan et al., 2022) or a mouse Crx-mCherry cassette (Gagliardi et al., 2018) into the *AAVS1* locus. A rod reporter line was made by replacing the *NRL* coding sequence with EGFP (Phillips et al., 2018); cone reporter lines were produced by inserting a mouse cone-arrestin (mCar)-GFP cassette (Gasparini et al., 2022) or inserting T2A-mCherry at the C terminus of *GNGT2* (Nazlamova et al., 2022); and a retinal ganglion cell (RGC) reporter line was produced by inserting a P2A-tdTomato-P2A-Thy1.2 cassette at the C-terminus of *BRN3B* (*POU4F2*) (Sluch et al., 2017). Additional lines reporting multiple cell types include a *SIX6*-GFP/*BRN3B*-tdTomato double reporter line that separately labels all retinal cells and RGCs, and a *VSX2*-Cerulean/*BRN3B*-EGFP/*RCVRN*-mCherry triple reporter line that differentially labels retinal progenitor cells, RGCs and photoreceptors (Lam et al., 2020; Wahlin et al., 2021). These lines have been used to investigate retinal morphogenesis, improve organoid differentiation, and purify specific retinal cell types for transcriptome profiling and cell transplantation.

To build a platform with which to study cone development and diseases, we sought to generate a human cone reporter iPSC line in which EGFP is specifically expressed in both immature and mature cones with minimal disruption of normal cone development. As a candidate, we identified guanine nucleotide-binding protein subunit alpha transducin 2 (*GNAT2*), which encodes the cone-specific α-subunit of transducin, a G-protein that couples visual pigment opsin to the cone phototransduction cascade (Morris et al., 1997; Morris and Fong, 1993). Prior studies demonstrated that *Gnat2^−/−^* mice exhibit complete loss of cone phototransduction without changes in rod phototransduction or in cone or rod morphology (Ronning et al., 2018). In human retina, *GNAT2* expression is limited to cones, controlled by a cone-specific promoter, and initially induced following the early cone expression of *RXRG* and *THRB* but prior to expression of mature cone markers *ARR3*, *OPN1SW* or *OPN1LW* (Hoshino et al., 2017; Morris et al., 1997; Welby et al., 2017) (Fig. S1A; data from Hoshino et al., 2017). However, unlike *RXRG* or *THRB*, *GNAT2* does not downregulate in adult cones (Hoshino et al., 2017; Welby et al., 2017). A similar onset order was observed in *CRX-*GFP^+^ photoreceptors in human ROs, with *GNAT2* first detected at day (d)37 (Kaewkhaw et al., 2015). These features suggested that the endogenous *GNAT2* promoter linked to a fluorescent protein could serve as an ideal cone-specific reporter.

In principle, cell type-specific fluorescent reporter lines enable live imaging to characterize developmental and disease processes. Episodic imaging (i.e. repeated imaging at defined intervals) of specific RO regions may enable long-term monitoring of individual retinal cells, yet embedding beyond the initial 6 weeks was shown to impair photoreceptor development (Decembrini et al., 2020; Rashidi et al., 2022). Others have immobilized and imaged mature ROs for up to 3 weeks (Achberger et al., 2019), but longer-term imaging has not been demonstrated.

In this study, we produced GNAT2-EGFP cone reporter iPSC lines in which cones are robustly, specifically and innocuously labeled with EGFP. We also established RO hydrogel immobilization and episodic live imaging methods that enable long-term assessment of individual EGFP^+^ cone morphological changes, inner segment development and mitochondria localization. This EGFP-GNAT2 cone reporter line, combined with the immobilization and imaging techniques, provides a useful tool to study cone development and disease.

## RESULTS

### GNAT2-EGFP iPSCs

To assess the suitability of a *GNAT2* cone reporter, we compared *GNAT2* expression to that of other potential cone markers in human fetal, adult and RO single-cell RNA-sequencing (scRNA-seq) datasets. In a combined human fetal retina, adult retina and early-stage RO dataset produced via 3′ end-counting, *GNAT2* was mainly detected in cones from adult retina (Fig. S1B-E; data from Lu et al., 2020). In contrast, our deep, full-length scRNA-seq analysis of fetal retinal progenitor cells and photoreceptors showed robust and specific *GNAT2* expression in the majority of fetal cones from post-conception week 13 to 19 (Fig. S1F,G; data from Shayler et al., 2023 preprint), consistent with the onset timing in bulk RNA-seq (Fig. S1A). This sensitivity and specificity compared favorably with that of other potential cone markers, such as *THRB*, *RXRG*, *GNGT2*, *ARR3*, *OPN1SW* or *OPN1LW*, which were either expressed in both cones and rods (*THRB*, *RXRG*, *GNGT2*; although the *THRB* TRβ2 isoform was cone specific) or expressed in only a subset of cones (*THRB* only in L/M cones, *ARR3* mainly in more mature cones, and *OPN1SW* and *OPN1LW* mainly in S and L/M cones, respectively) (Fig. S1H-O; data from Shayler et al., 2023 preprint). These patterns were preserved in human ROs, in which *GNAT2* was first detected at d37 in CRX-GFP positive cells (Kaewkhaw et al., 2015). These analyses supported the potential utility of a *GNAT2*-driven cone reporter.

**Fig. 1.**
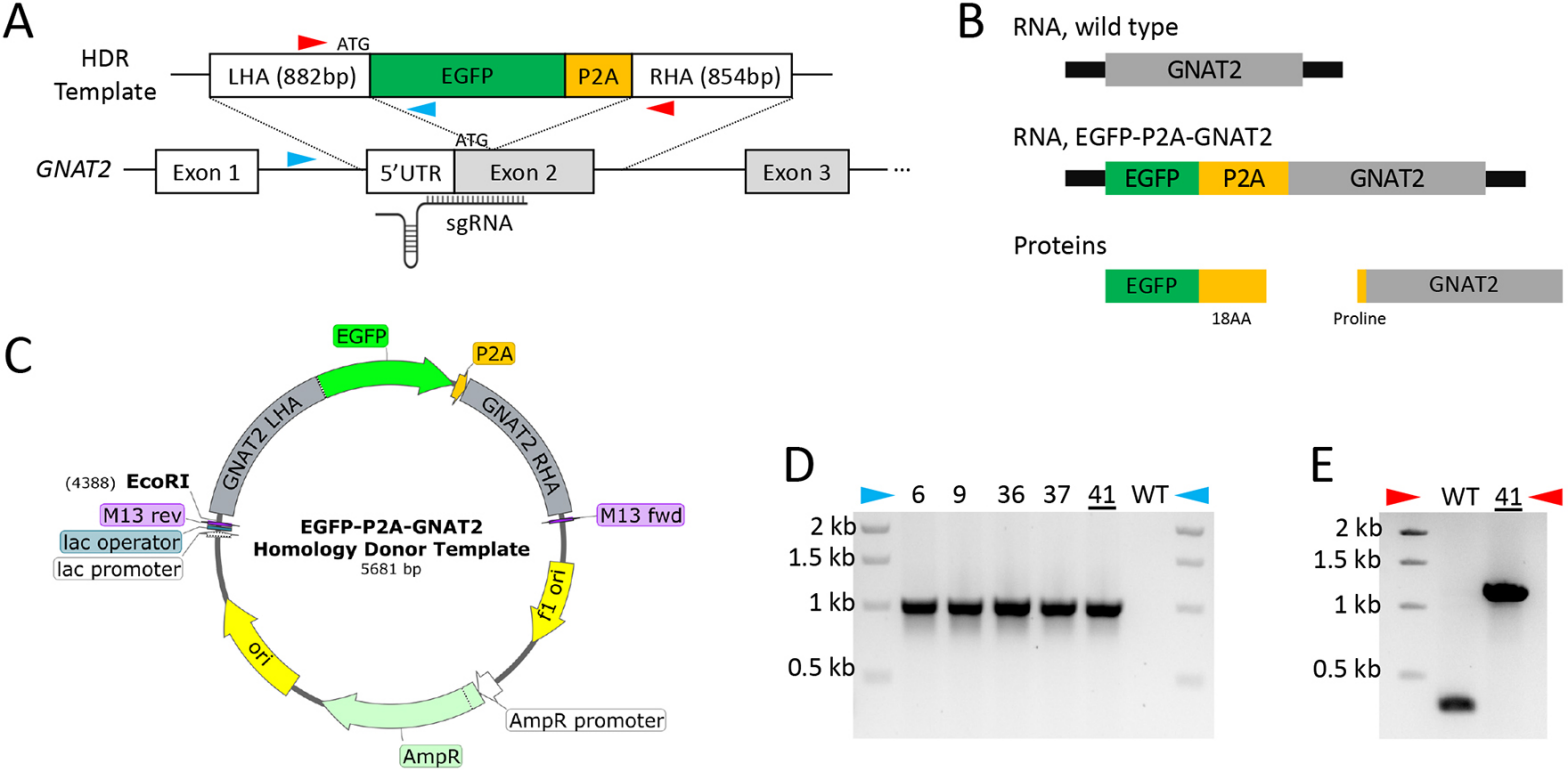
**Generation of GNAT2-EGFP induced pluripotent stem cells (iPSCs).** (A) Strategy for EGFP-P2A knock-in at the *GNAT2* N-terminus. The EGFP-P2A cassette is inserted after the endogenous *GNAT2* ATG start codon. The sgRNA spans the knock-in junction. LHA, left homology arm; RHA, right homology arm. Blue arrowheads indicate the location-specific genotyping primers. Red arrowheads indicate the insert-flanking genotyping primers. (B) Schematic of RNA and protein expressed by wild-type and *GNAT2-EGFP* alleles. After translation and P2A cleavage, 18 P2A amino acid residues are added to the C-terminus of EGFP while a proline residue is added to the N-terminus of GNAT2. (C) Homology donor template map with EGFP-P2A cassette flanked by GNAT2 LHA and RHA. (D) Genotyping PCR with location-specific primer pairs. The 1 kb bands in clone (C)-6, C-9, C-36, C-37 and C-41 indicate knock in of the EGFP-P2A cassette with correct orientation. (E) Genotyping PCR with insert flanking primer pairs. The presence of 1.2 kb band and absence of 0.3 kb band in C-41 indicate bi-allelic knock-in.

To generate a *GNAT2* cone reporter line, we used CRISPR/Cas9-mediated homologous recombination to insert an EGFP-P2A cassette at the N-terminus of *GNAT2* in the human WTC11 iPSC-derived line WTC11-mTagRFPT-LMNB1 (Allen Institute for Cell Science) (Fig. 1A,B). The N-terminal position of the EGFP-P2A cassette is predicted to enable GNAT2 translation with a single proline residue added to the N-terminus (Fig. 1B). WTC11-mTagRFPT-LMNB1 cells and derivatives express an mTagRFPT-LMNB1 fusion protein that labels nuclear envelopes and enables live imaging of nuclei together with other fluorescent protein markers.

Briefly, a homology donor plasmid was constructed by inserting the EGFP-P2A coding sequence between left and right homology arms (LHA and RHA, respectively) containing human *GNAT2* genomic sequences 882 bp upstream and 854 bp downstream of the translation start codon (Fig. 1C). The sgRNA spanned the intended insertion site, eliminating the need to introduce a silent mutation on the homology donor plasmid (Fig. 1A). No antibiotic resistance marker was included in the donor vector to enable scarless editing. Following electroporation of the donor plasmid and a plasmid co-expressing *GNAT2* sgRNA and Cas9-T2A-Puro (PX459) (Ran et al., 2013), cells were selected with puromycin, single-cell cloned, and screened by PCR using location-specific and insert-flanking primer pairs (Fig. 1A). PCR with location-specific primers flanking the LHA showed integration of the EGFP-P2A cassette with correct orientation in five of 48 clones tested (Fig. 1D), while insert-flanking primers distinguished two mono-allelic from three bi-allelic knock-in clones (Fig. 1E and data not shown). The two mono-allelic clones carried mutations on the non-knock-in alleles, whereas the three bi-allelic clones carried no unintended mutations at the knock-in junctions. Partial sequencing of EGFP-P2A-GNAT2 (hereafter referred to as GNAT2-EGFP) clone (C)-41 revealed no unintended mutations at any of the top five predicted gRNA off-target sites (Fig. S2A,B, Table S1), and karyotyping revealed no chromosomal abnormalities (Fig. S2C). All subsequent experiments were carried out using this clone.

### GNAT2-EGFP ROs

**Fig. 2.**
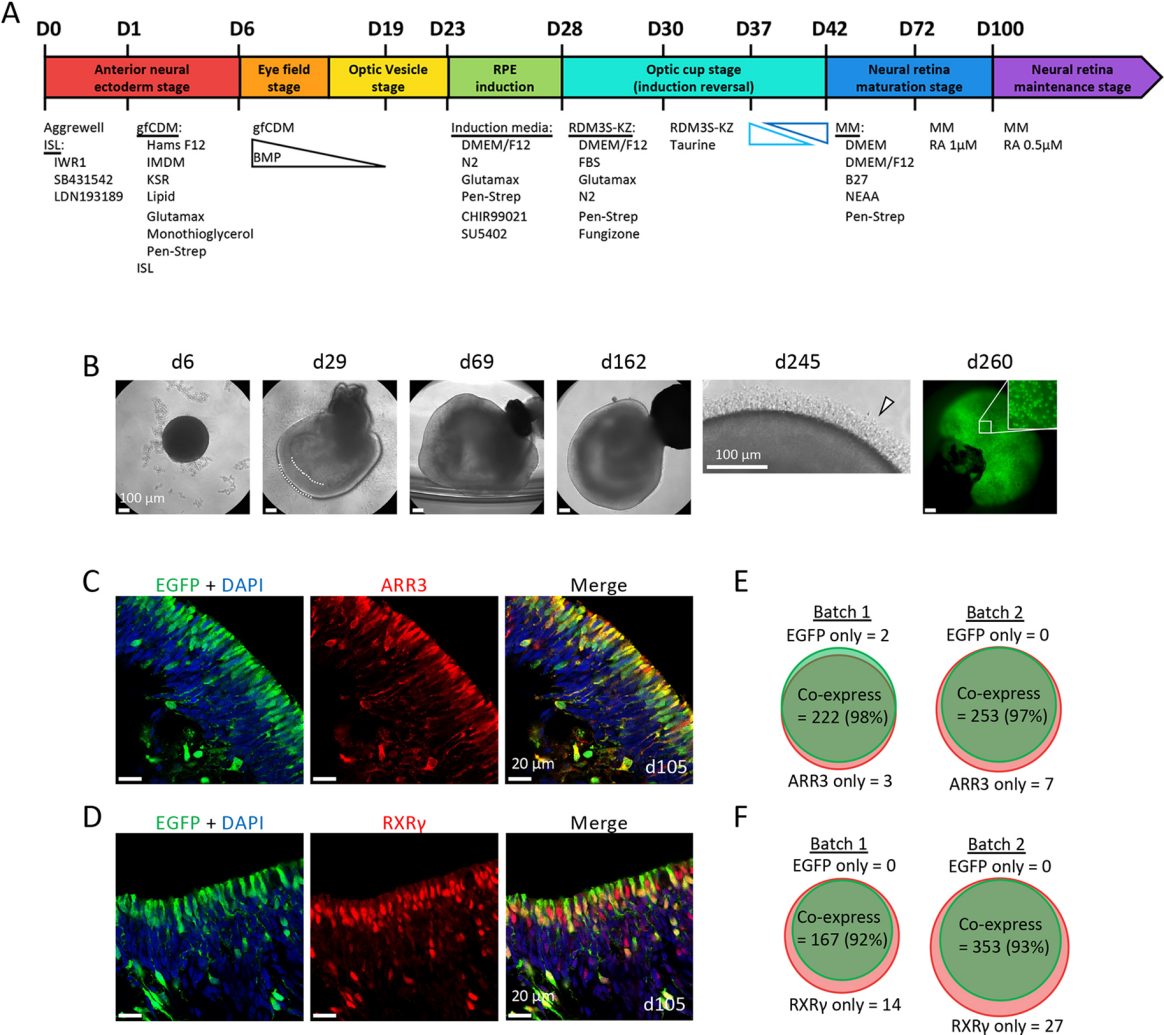
**Generation and characterization of GNAT2-EGFP retinal organoids (ROs).** (A) Overview of the RO differentiation protocol. D, day; MM, maintenance medium; RA, retinoic acid; RPE, retinal pigment epithelium. (B) Representative phase-contrast images of GNAT2-EGFP ROs at day (d)6, d29, d69, d162 and d245, and fluorescent image at d260. White dotted lines in the d29 image indicate presumptive developing neural retina. Arrowhead in the d245 image indicates a visible brush border on a mature RO. Scale bars: 100 µm. (C,D) Representative immunostaining of d105 GNAT2-EGFP RO, indicating co-expression of EGFP and ARR3 (C) or EGFP and RXRγ (D). Scale bars: 20 µm. (E) Quantification of cells expressing EGFP only, ARR3 only or both in nine sections from four ROs, two independent differentiations. (F) Quantification of cells expressing EGFP only, RXRγ only or both in eight sections from four ROs, two independent differentiations.

We next evaluated the ability of the GNAT2-EGFP iPSCs to make ROs with cone-specific EGFP expression. We used a modified version of the protocol by Kuwahara et al. (2015) to improve RO consistency. Initial culture medium was supplemented with small-molecule inhibitors of WNT signaling (IWR1) and TGF-β superfamily signaling (SB431542 and LDN193189) for 6 days, followed by addition of BMP-4 to induce anterior neural ectoderm and eye field specification (Aparicio et al. 2023). As the parental WTC11-mTagRFPT-LMNB1 had poor early survival, the starting cell number was increased from 12,000 to 48,000, the retinal pigment epithelium induction reversal was optimally timed from d23 to d28, and long-term maintenance with retinoic acid was begun at d72 (Fig. 2A), which promotes photoreceptor maturation and long-term survival (Kelley et al., 1994; Zhong et al., 2014). During the first 30 days, ROs increased in size and adopted a structure consistent with that of nascent neural retina (Fig. 2B). Subsequently, ROs continued to grow, and a brush border likely representing photoreceptor inner and/or outer segments was evident by ∼d140 and remained visible until the latest analysis on d245 (Fig. 2B). In mature ROs, EGFP^+^ cells formed uneven patches occupying the outermost layer (Fig. 2B).

To evaluate the specificity of GNAT2-EGFP expression, we immunostained d105 RO sections from two independent differentiations with cone markers ARR3 and RXRγ and assessed their colocalization with EGFP. At this age, most EGFP^+^ cells had elongated cell bodies occupying the outermost layer (Fig. 2C,D). Among 487 cells examined for ARR3 and EGFP, 475 (97.5%) co-expressed both, ten expressed ARR3 only, and two expressed EGFP only (Fig. 2C,E). Among 561 cells examined for RXRγ and EGFP, 520 (92.7%) co-expressed both, 41 expressed RXRγ only, and none were EGFP^+^ and RXRγ^−^ (Fig. 2D,F, unpaired two-tailed Student's *t*-test, *P*=0.019, for % EGFP^+^,ARR3^+^ versus EGFP^+^,RXRγ^+^). Similarly, at d86, all EGFP^+^ cells expressed RXRγ and photoreceptor marker CRX (Fig. S3A-D). Moreover, 35.2% of RXRγ^+^ cells were EGFP^−^ at d86 (Fig. S3C) compared to 7.3% at d105 (Fig. 2F, unpaired two-tailed Student's *t*-test, *P*=0.02). These results are consistent with the sequential onset of RXRγ, GNAT2-EGFP and ARR3 expression during cone maturation (Hoshino et al., 2017; Welby et al., 2017) (Fig. S1A).

Additionally, immunostaining of GNAT2-EGFP ROs for retinal progenitor cell markers PAX6 and CHX10 (VSX2), RGC marker BRN3B (POU4F2), bipolar cell marker CHX10, amacrine cell marker AP2α (TFAP2A), rod marker NRL, Müller glia marker CRALBP (RLBP1) and horizontal cell marker calbindin, at various ages, showed the presence of all retinal cell types and no co-expression of EGFP (Fig. S3E-L). Although most retinal cells resided in their expected RO layers, some misplaced EGFP^+^,RXRγ^+^ and EGFP^+^,ARR3^+^ cones resided deep within the ROs, as reported by others (Capowski et al., 2019; Wahlin et al., 2017). Taken together, these results confirm the specific and robust labeling of cones in GNAT2-EGFP ROs.

To evaluate the effects of the EGFP-P2A tag on GNAT2 expression, western blot analysis of ROs derived from two independent differentiations of GNAT2-EGFP C-41 and parental WTC-mTagRFPT-LMNB1 iPSCs was performed. The results showed that GNAT2-EGFP ROs had a slight, but not statistically significant, decrease in GNAT2 expression and no change in GNAT2 size (Fig. S3M,N).

### High-resolution live confocal imaging of cone maturation

**Fig. 3.**
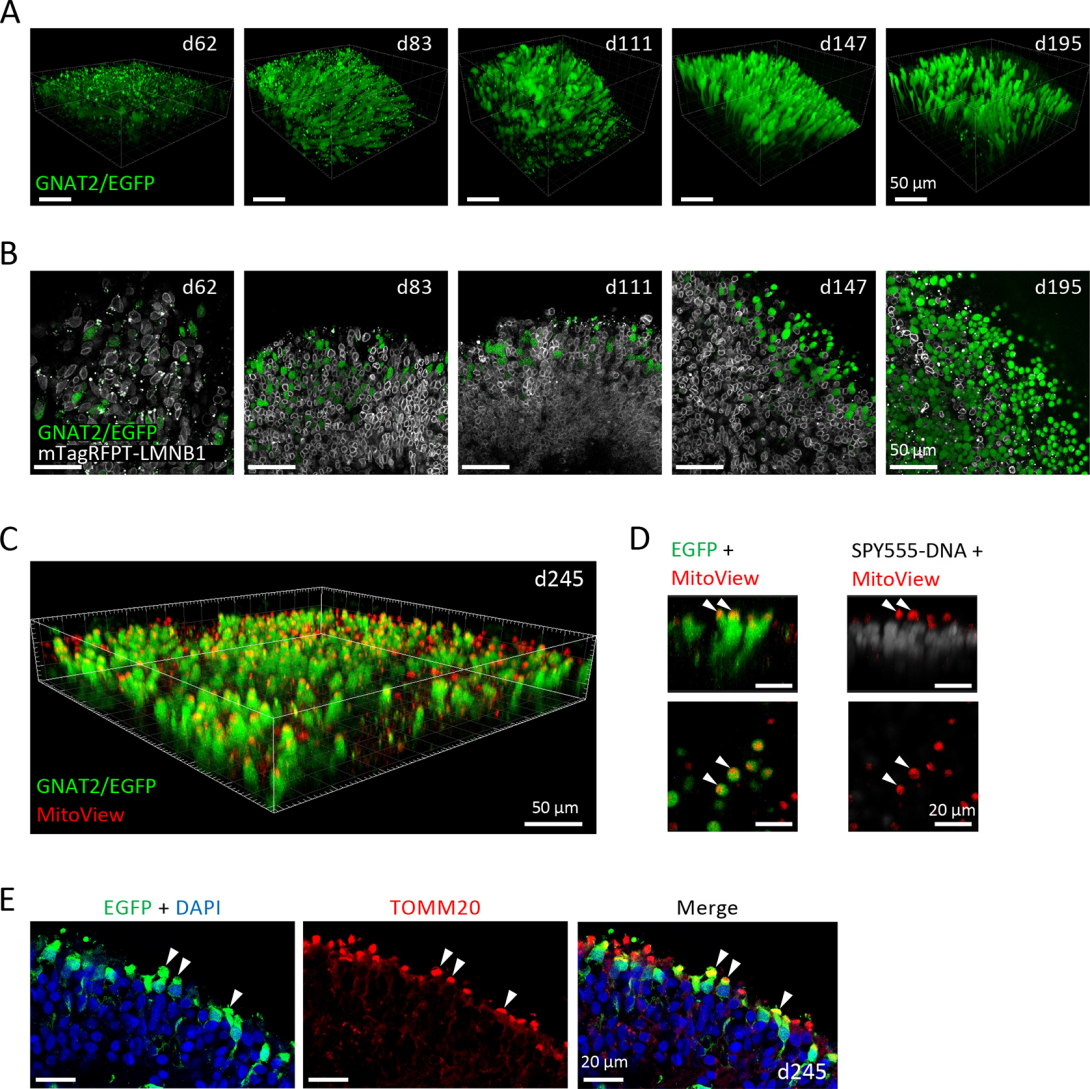
**Live confocal imaging of GNAT2-EGFP ROs.** (A) Representative 3D renderings of *z*-stack live confocal images of GNAT2-EGFP ROs at d62, d83, d111, d147 and d195. EGFP^+^ cells adopted mature cone morphology in late time points (d147 and d195). Scale bars: 50 µm. (B) Single *z*-section from the *z*-stack images from A in which nuclei are marked by mTagRFPT-LMNB1 and cones are marked by EGFP. Scale bars: 50 µm. (C) Representative 3D rendering of live confocal *z*-stack images of d245 GNAT2-EGFP RO incubated with MitoView. Scale bar: 50 µm. (D) Cross-section images from the *z*-stack image in C, showing coalescence of mitochondria at the EGFP^+^ cone inner segments above the nuclear layer stained with SPY555 DNA (arrowheads). (E) Immunostaining of d245 GNAT2-EGFP RO, confirming coalescence of TOMM20^+^ mitochondria with EGFP^+^ cone inner segments (arrowheads). Scale bars: 20 µm.

We next assessed the feasibility of high-resolution live imaging of GNAT2-EGFP ROs to monitor the development of cone cells over time. We performed episodic live confocal imaging on GNAT2-EGFP ROs and captured *z*-stack images at different maturation stages, with cones represented by cytoplasmic+nuclear EGFP and nuclear membranes represented by mTagRFPT-LMNB1 (Fig. 3A,B). The cytoplasmic+nuclear EGFP distribution is consistent with P2A-mediated separation of EGFP from GNAT2 and the ability of EGFP to diffuse across the nuclear envelope (Seibel et al., 2007; Wei et al., 2003). From d62 to d111, EGFP^+^ cell bodies elongated and gradually populated the outermost organoid layer. By d147, they developed more mature cone morphology, with the appearance of inner segments and pedicles similar to those in older ROs. By d195, cones retained similar morphology, and cell bodies were often displaced away from the outermost layer as previously described (Gasparini et al., 2022).

Unexpectedly, the live-imaged mTagRFPT-LMNB1 nuclear envelope signal was much weaker in EGFP^+^ cones than in EGFP^−^ cells (Fig. 3B; Fig. S4A). Similarly, immunostaining of d160 GNAT2-EGFP ROs and d105 ROs differentiated from the parental WTC-mTagRFPT-LMNB1 iPSCs showed decreased LMNB1 in EGFP^+^ and RXRγ^+^ cone nuclei (Fig. S4B,C). The decreased cone LMNB1 expression is unlikely to relate to the heterozygous mTagRFPT knock-in allele as the wild-type *LMNB1* allele remains. These analyses confirm that live imaging can be used to display the distribution of EGFP^+^ cones and mTagRFPT^+^ nuclei.

Live confocal imaging also enabled assessment of organelle development. At d245, imaging with MitoView dye, which accumulates in mitochondria and allows live detection, showed that mitochondria coalesced at the mature cones' inner segment ellipsoid bodies (Fig. 3C,D), as confirmed by immunostaining with the mitochondrial marker (TOMM20) (Fig. 3E).

### Cone maturation in hydrogel-immobilized GNAT2-EGFP ROs

**Fig. 4.**
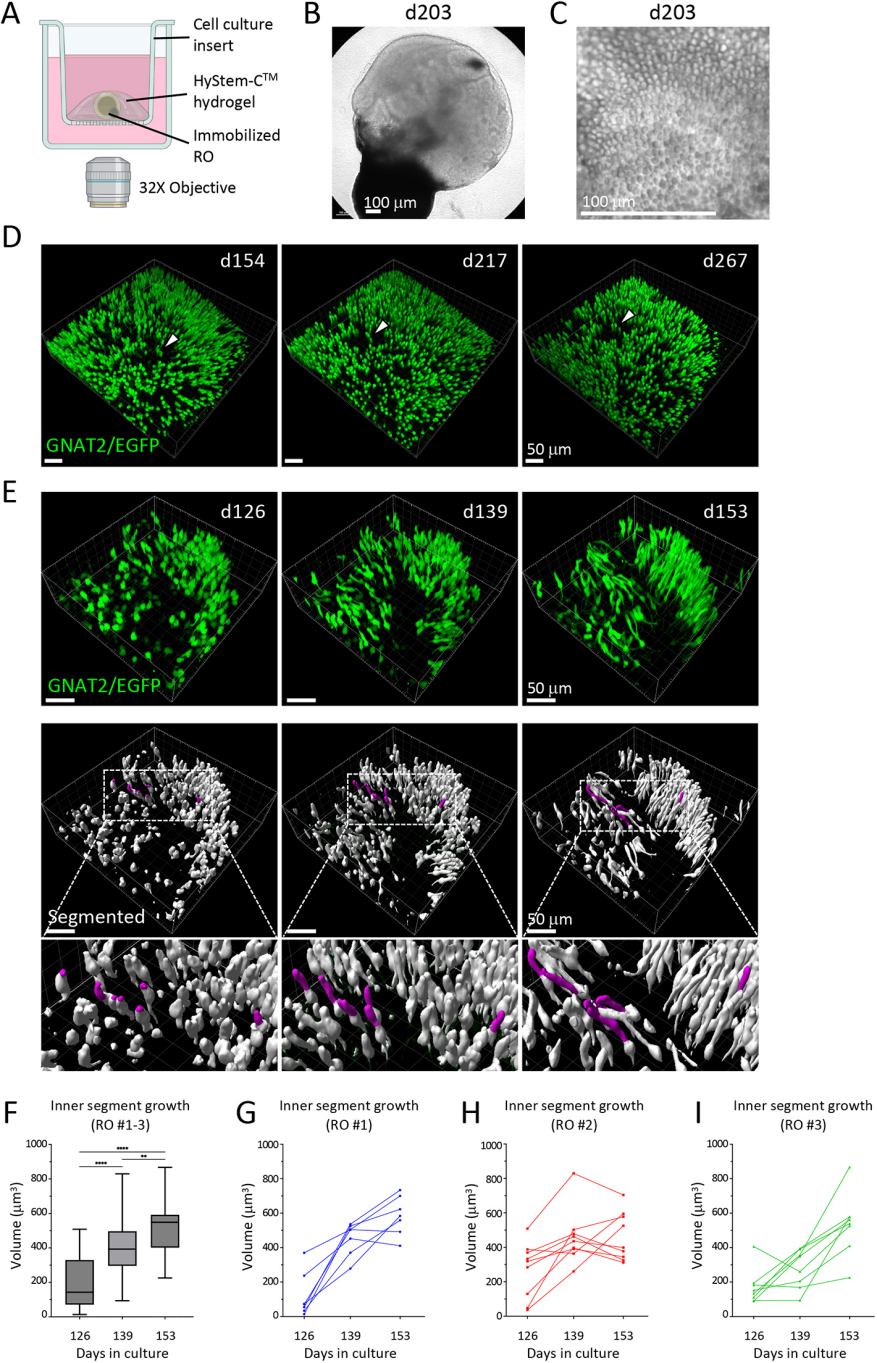
**Live confocal imaging of hydrogel-embedded GNAT2-EGFP ROs.** (A) Schematic of RO embedding and imaging in hydrogel on Millicell^TM^ cell culture insert (created with BioRender.com). ROs are submerged under medium and imaged through the bottom of the dish. (B) Phase-contrast image of a d203 RO embedded in hydrogel. Scale bar: 100 µm. (C) Phase-contrast image of a hydrogel-embedded d203 RO showing visible photoreceptor inner segment protrusion on the RO surface. Scale bar: 100 µm. (D) Representative 3D renderings of *z*-stack live confocal images of hydrogel-embedded GNAT2-EGFP ROs at d154 (15 days in hydrogel), d217 (78 days in hydrogel) and d267 (128 days in hydrogel). Arrowheads indicate the same group of three cones at each imaging time point. Scale bars: 50 µm. (E) Representative 3D renderings of *z*-stack live confocal images of hydrogel-embedded GNAT2-EGFP ROs at d126, d139 and d153 (top panel), and the EGFP intensity and cell shape-based segmentation results (bottom panel). EGFP^+^ cone inner segment development was tracked. Magenta surfaces indicate the inner segments of seven cones tracked and quantified for RO #1. Scale bars: 50 µm. (F) Pooled quantification of the inner segment volumetric change from 24 cones on three ROs from d126 to d153. Boxes represent the 25-75th percentiles, and the median is indicated. The whiskers indicate the range. **P=0.0062; ****P<0.0001 (one way ANOVA). (G-I) Inner segment volumetric change of individual cones from d126 to d153.

To evaluate the maturation of individual cone cells, ROs were embedded in HyStem-C^TM^ hydrogel on Millicell^TM^ cell culture inserts, and the same organoid regions were repeatedly imaged during long-term culture (Fig. 4A). HyStem-C^TM^ is based on hyaluronic acid and collagen crosslinked polymers and was chosen because its rigidity can be tuned, and because hyaluronic acid and collagen are major components of retinal extra cellular matrix and vitreous humor (Achberger et al., 2019; Hemshekhar et al., 2016; Tram and Swindle-Reilly, 2018) and deemed likely to be biocompatible. Pilot experiments showed that 0.25-1% HyStem-C^TM^ supported long-term immobilization, whereas organoids dislodged from 2% and 4% hydrogel. ROs embedded in 1% hydrogel at d121 or older could be cultured in this manner for at least 120 more days. The embedded ROs displayed continuous morphological changes with the appearance of photoreceptor inner segment protrusions, suggesting that embedding does not restrict cone growth and maturation (Fig. 4B,C). Indeed, in organoids embedded on d139, mature cones remained stable and individual cells were identifiable until at least d267 (18.3 weeks) (Fig. 4D).

Episodic live confocal imaging of immobilized ROs allowed us to capture developmental features of the same cells over time. As an example, we used EGFP intensity and cell shape-based segmentation on the acquired three-dimensional (3D) *z*-stack images to define the volumetric change in 24 cone inner segments in three ROs immobilized at d125 and imaged between d126 and d153 (Fig. 4E). The individual cells were within a defined region in which spatial relationships were maintained. We observed the inner segments enlarged from a mean of 193 µm^3^ to 523 µm^3^ (*P*<0.0001) (Fig. 4F), with an average increment of 12.2 µm^3^ per day. However, the initial inner segment size and rate of individual cone inner segment growth varied depending on the organoid and/or organoid region, ranging from a maximum rate of 27.14 µm^3^ per day for one cone from RO #3 to a slight decline of 0.3 µm^3^ per day for another from RO #2 (Fig. 4G-I). Taken together, episodic live imaging of hydrogel-embedded GNAT2-EGFP ROs enabled long-term evaluation of cone development, such as inner segment morphogenesis and formation of mitochondria-rich ellipsoid bodies, demonstrating the versatility of this reporter system.

## DISCUSSION

In this study, we generated a cone-specific GNAT2-EGFP iPSC reporter line and demonstrated its utility for tracking individual cone development in long-term live-embedded ROs. By tagging *GNAT2* with scarless CRISPR insertion and placing the EGFP-P2A at the N-terminus, we aimed to create a reporter line that faithfully recapitulates cone development with minimal effect on *GNAT2* expression or protein sequence. The GNAT2-EGFP iPSC line generates all retinal cell types and robustly labels GNAT2^+^ cones throughout RO differentiation, with EGFP detected in cone precursor cell bodies at d62 and subsequently in maturing cone axon terminals, nuclei and inner segments. Indeed, EGFP was detected as early as d34, when RO cone precursors are initially born (Kaewkhaw et al., 2015). However, as cone-specific genes might transiently express before cell fate is determined, lineage tracing is needed to determine whether the earliest GNAT2-EGFP expression is strictly limited to the cone photoreceptor lineage.

Live imaging revealed that maturing cone precursors develop inner segments and extend pedicles towards the outer plexiform layer between ∼d120 and ∼d150, coinciding with a time of rapid maturation and high glycolytic activity in the photoreceptor layer (Browne et al., 2017). This timeline is slightly delayed compared to that of foveal cones in the human fetal retina, which elongate and develop inner segments and synaptic pedicles between fetal week 14 and 18 (d98-d126) (Hendrickson and Zhang, 2019), but is similar to that of the later-born peripheral cones that RO cone transcriptomes best resemble (O'Hara-Wright and Gonzalez-Cordero, 2020).

Recently, two other cone reporter human iPSC lines have been described – one generated by piggyBac-mediated insertion of GFP under the control of mCar promoter (Gasparini et al., 2022) and the other generated by inserting a T2A-mCherry cassette into the *GNGT2* locus (Nazlamova et al., 2022). Cone-arrestin is first expressed at a later stage of cone maturation than GNAT2, limiting its ability to label immature cones (Hoshino et al., 2017; Welby et al., 2017) (Fig. S1A). Moreover, in mature ROs, only ∼80% of cells labeled with the mCar-GFP reporter were ARR3^+^ whereas >95% were recoverin^+^ (Gasparini et al., 2022), potentially reflecting some non-cone expression. Our CRISPR-based editing of *GNAT2* is similar to the Nazlamova et al. (2022) editing of *GNGT2*, but used an antibiotic-free scarless knock-in approach so the inserted sequence contains only EGFP-P2A. Additionally, 3′ end counting and our full-length scRNA-seq analyses indicate that *GNAT2* RNA is highly cone specific, whereas *GNGT2* RNA is expressed in both cones and rods and has no discernable difference in cone- versus rod-specific splicing or exon usage (Fig. S1E,H,N,O) (Lu et al., 2020; Shayler et al., 2023 preprint). Nevertheless, transcript expression need not directly correlate with protein expression, as evidenced by the cone-specific GNGT2 protein in developing and adult human retinae (Ong et al., 1997) and the cone-specific mCherry protein in *GNGT2-T2A-mCherry* ROs (Nazlamova et al., 2022), suggesting that post-transcriptional mechanisms enforce cone-specific translation of GNGT2 and the linked mCherry in *GNGT2-T2A-mCherry* ROs, despite similar cone and rod *GNGT2* RNA levels.

Furthermore, we established a protocol with which to track individual cone development. Live embedding in HyStem-C^TM^ hydrogel enabled long-term immobilized RO cultures and monitoring of cone development with episodic live confocal imaging (i.e. repeated imaging at defined intervals). Immobilizing the ROs in thin layers of hydrogel allows nutrient diffusion from all sides and allows RO to grow and mature with minimal restriction (Fig. 4A). Cells were tracked for at least 120 days starting at >d120. The hydrogel maintained structural integrity and transparency during the entire period, allowing repeated imaging of the same RO regions. However, live embedding might be more disruptive in younger <d80 ROs owing to the ongoing rapid organoid growth and morphological changes. The biocompatibility of HyStem-C^TM^ hydrogel was evident from the fairly consistent growth of cone inner segments and may relate to its derivation from hyaluronic acid and collagen, which are major components of vitreous humor and retinal extracellular matrix (Achberger et al., 2019; Hemshekhar et al., 2016; Tram and Swindle-Reilly, 2018). Combining this GNAT2-EGFP cone reporter with further CRISPR editing and live imaging provides a powerful tool to study cone development and disease.

## MATERIALS AND METHODS

### Human iPSC culture

WTC-mTagRFPT-LMNB1 human iPSC line was obtained from Allen Institute for Cell Science (https://www.allencell.org/cell-catalog.html), cell line ID AICS-0034 cl.62. This line was produced as described in Roberts et al. (2017). The WTC-mTagRFPT-LMNB1 iPSCs and the subsequent GNAT2-EGFP iPSCs were cultured in feeder-free conditions in mTeSR Plus Medium (Stem Cell Technologies, 100-0276) on Matrigel (Corning, 354277)-coated 35 mm dishes. Cells were seeded at 25,000 cells per dish, fed daily and passaged every 5 days at ∼70% confluency. When passaged, cells were washed with DPBS (Corning, 21-031-CV), and colonies were gently lifted by 2 min incubation in ReLeSR (Stem Cell Technologies, 05872) at 37°C. After neutralization with mTeSR Plus Medium, cells were centrifuged for 3 min at 300 ***g*** and seeded at desired densities. WTC-mTagRFPT-LMNB1 iPSCs and GNAT2-EGFP iPSCs were authenticated and confirmed to be mycoplasma negative.

### Generation of EGFP-P2A-GNAT2 homology donor and sgRNA plasmids

The 882 bp LHA and 854 bp RHA were PCR amplified from WTC-mTagRFPT-LMNB1 iPSC genomic DNA with CloneAmp HiFi PCR Premix (Takara, 639298). The LHA, EGFP-P2A and RHA were cloned into pUC118 backbone using In-Fusion Snap Assembly (Takara, 638949). The sgRNA targeting the GNAT2 ATG^Met^ start codon (AAGACGGCAAATATGGGAAG) was identified using the CRISPick online sgRNA design tool (https://portals.broadinstitute.org/gppx/crispick/public) and cloned into the PX459 sgRNA Cas9-T2A-Puro expression plasmid (Addgene #62988) (Ran et al., 2013) according to the accompanying Zhang Laboratory Target Sequence Cloning Protocol (https://media.addgene.org/data/plasmids/62/62988/62988-attachment_KsK1asO9w4owD8K6wp8.pdf). The resulting plasmids were sequenced to confirm correct assembly. A full list of cloning primers can be found in Table S2.

### Generation of GNAT2-EGFP iPSCs

WTC-mTagRFPT-LMNB1 human iPSCs were dissociated into single cells with 5 min incubation in Accutase (Life Technologies, A1110501) at 37°C. Then, 200,000 cells were electroporated with 500 ng PX459 and 1000 ng homology donor plasmid in 10 µl electroporation buffer R using a Neon Transfection System (Invitrogen) with one pulse of 1400 V and a pulse width of 20 ms. After electroporation, cells were placed in mTeSR Plus Medium supplemented with CloneR (Stem Cell Technologies, 05888) for 36 h and then selected in 250 ng/ml puromycin for 36 h. After a recovery phase of 4 days, colonies were dissociated into single cells with Accutase and seeded into 96-well plates at an average density of 0.5 cells per well to ensure that colonies are derived from single cells. Colonies were expanded and genotyped using two primer pairs: an LHA flanking primer pair that produces a 990 bp band from clones with correct integration of the insert, and an insert flanking primer pair that produces a single 1131 bp band from bi-allelic knock-in clones, the 1131 bp band and a 351 bp from mono-allelic knock-in clones, or a single band at 351 bp from wild-type clones. The 1131 bp knock-in band and 351 bp wild-type allele band were gel purified (Qiagen, 28604) and sequenced to check for potential mutations introduced during editing. To check for potential off-target mutations, we PCR amplified ∼1 kb regions spanning the top five program predicted off-target sites (IDT CRISPR-Cas9 gRNA checker) from the edited clone-41 and the unedited WTC-mTagRFPT-LMNB1 iPSCs and aligned the sequences. All PCRs utilized CloneAmp HiFi PCR Premix according to the manufacturer's instructions. The predicted off-target sequences and amplifying primers are provided in Tables S1 and S2. The GNAT2-EGFP iPSC line was karyotyped by the Department of Pathology and Laboratory Medicine, Children's Hospital Los Angeles.

### Retinal organoid differentiation

Retinal organoids were generated following steps modified from a previous protocol (Kuwahara, et al., 2015; Aparicio et al. 2023). Briefly, iPSCs were dissociated on d0 with Accutase and resuspended in Aggrewell media (Stem Cell Technologies, 05893) supplemented with 20 µM Y-27632 (Cayman Chemical, 10005583) and ‘ISL’ cocktail, which consists of 3 µM IWR1 (Cayman Chemical, 13659), 10 µM SB431542 (Cayman Chemical, 13031), and 0.1 µM LDN193189 (Sigma-Aldrich, SML0559). Cells were plated in round-bottom 96-well plates at 48,000 cells per well in 200 µl medium to allow aggregate formation. From d1 to d5, medium was changed to gfCDM [45% Hams F12 (Thermo Fisher Scientific, 11765047), 45% IMDM (Thermo Fisher Scientific, 12440046), 10% KSR (Life Technologies, 10828028), 1× Chemically Defined Lipid (Gibco, 11905-031), 0.5× Glutamax (Life Technologies, 35050061), 450 µM monothioglycerol (Sigma-Aldrich, M6145) and 1× Penicillin-Streptomycin (Corning, 30-002-CI)] supplemented with ISL. On d6, gfCDM medium was supplemented with 0.75 nM BMP-4 (R&D Systems, 314-BP-050), followed by 1/2 medium change on d9 and d12, 3/4 medium change on d15, and full medium change on d19 and d21 with fresh gfCDM only without BMP-4. From d23 to d27, medium was changed to RPE induction medium that consisted of Dulbecco's modified Eagle medium (DMEM)/F12 (Thermo Fisher Scientific, 21331020), 1× N2 supplement (Thermo Fisher Scientific, 17502048), 1× Glutamax, 1× Penicillin-Streptomycin, 3 µM CHIR99021 (Cayman Chemical, 13122) and 5 µM SU5402 (Cayman Chemical, 13182). Starting at d28, medium was changed to RDM3S-KZ, which consisted of DMEM/F12, 10% fetal bovine serum (FBS; Omega Scientific, FB-01), 1× Glutamax, 1× N2 supplement, 1× Penicillin-Streptomycin and 0.5× fungizone (Omega Scientific, FG-70). Taurine (Sigma-Aldrich, T8691; 0.1 mM) was added from d30 onward. On d30, ROs were transferred to a 48-well cell culture plate pre-coated with HEMA (Sigma-Aldrich, P3932-25G). From d37 to d42, medium was transitioned to RO maintenance medium (MM) adapted from Zhong et al., (2014): 2/3 RDM3S-KZ+1/3 MM on d37, 1/3 RDM3S-KZ+2/3 MM on d40, and all MM on d42. MM consisted of an equal volume of DMEM (VWR, 54000-305) and DMEM/F12, supplemented with 1× B27 supplement (Thermo Fisher Scientific, 12587010), 1× NEAA, 1× Penicillin-Streptomycin, 1× fungizone, 10% FBS, 0.1 mM taurine and 1× Glutamax. ROs were subsequently cultured in MM with 1 µM retinoic acid (Sigma-Aldrich, R2625) from d72 to d100 and 0.5 µM retinoic acid from d100 onward. Differences from the protocol of Aparicio et al. (2023) included the following: (1) cultures were initiated with 48,000 cells; (2) only BMP-4 was added on d6, no IWR1; (3) induction reversal was initiated on d23 for 5 days; and (4) RA was first added on d72.

### Immunofluorescent staining and quantification

Samples comprised of four to six ROs were fixed in 4% paraformaldehyde for 12 min, washed with DPBS three times, incubated in 30% sucrose solution overnight at 4°C, embedded in OCT compound and cryo-sectioned into 20 µm sections. For immunostaining, slides were washed with TBS, blocked for 1 h at room temperature (2.5% horse serum, 2.5% donkey serum, 2.5% human serum, 1% bovine serum albumin, 0.1% Triton X-100 and 0.05% Tween 20 in 1× TBS), incubated in primary antibodies at 4°C overnight, followed by TBS wash, secondary antibody and 4′,6-diamidino-2-phenylindole (DAPI) incubation at room temperature for 1 h, washing and mounting with Mowiol with anti-fade. Samples were imaged on Zeiss LSM710 or Leica Stellaris 5 confocal microscopes and processed using Fiji ImageJ's Cell Counter plugin. ROs from three independent differentiation experiments were collected at different ages, and multiple sections from at least two ROs from each differentiation were analyzed. Primary antibodies are listed in Table S3.

### Western blotting and quantification

Samples comprised of eight to ten ROs at d70 were dissociated with 10 U/ml Papain (Worthington Biochemical, LK003176) and 100 U/ml DNaseI (Qiagen, 79254) in Earle's balanced salt solution at 37°C for 30 min, with periodic trituration using a P1000 pipette to aid dissociation. Dissociated ROs were washed with PBS, lysed with RIPA buffer (Cell Signaling Technology, 9806S) plus a protease inhibitor cocktail (Sigma-Aldrich, 5892970001) for 1 h on ice, and centrifuged at 15,000 ***g*** at 4°C for 10 min. Supernatants were collected, and proteins were quantified via bicinchoninic acid (BCA) assay. Lysates containing 50 µg total protein were mixed with 4× sample buffer (Invitrogen, NP0007), boiled at 95°C for 5 min, separated on SDS-polyacrylamide gel (Invitrogen, NP0335BOX) and transferred to a PVDF membrane (Sigma-Aldrich, GE10600023). After blocking with 5% milk and 0.05% Tween 20 in 1× TBS, the membrane was sequentially incubated with anti-GNAT2 primary antibody (Thermo Fisher Scientific, PA5-22340) and horseradish peroxidase (HRP)-conjugated anti-rabbit IgG (Cell Signaling Technology, 7074P2). Protein bands were detected using SuperSignal™ Ultimate Sensitivity Chemiluminescent Substrate (Thermo Fisher Scientific, A38555). After imaging, the membrane was stripped with stripping buffer (Thermo Fisher Scientific, 46430) for 15 min, re-blocked with milk and incubated with anti-β-actin primary (Cell Signaling Technology, 4967S) and HRP-conjugated secondary antibodies (for antibody dilutions, see Table S3). Fiji ImageJ was used to quantify protein bands, and GraphPad Prism was used to perform unpaired two-tailed Student’s *t*-test and to generate the graph.

### RO immobilization, live episodic confocal imaging and image processing

ROs were subjected to live episodic imaging using a Zeiss LSM780 NLO inverted confocal microscope, with fitted temperature control and a CO_2_ chamber that kept ROs at 37°C and 5% CO_2_ during imaging. A Zeiss C-Achroplan 32×/0.85 W Korr M27 lens (working distance 1.1 mm) was used to accommodate the long distance between the lens and ROs embedded in cell culture inserts. Non-immobilized ROs were submerged in RO medium and imaged in a Lab-Tek eight-well chambered coverglass (Thermo Fisher Scientific, 12-565-338). For mitochondria live imaging, ROs were incubated with 1× SPY555 vital DNA dye (Spirochrome, SC201) overnight and MitoView 650 (Biotium, 70075) at 200 nM for 30 min prior to imaging. For RO immobilization, individual ROs were live embedded in 100 µl 1% HyStem-C^TM^ hydrogel (Advanced Biomatrix, GS312) on a Millicell^TM^ 12 mm cell culture insert with 0.4 µm hydrophilic PTFE membrane (Sigma-Aldrich, PICM01250). The cell culture insert was then submerged in RO medium in a 24-well plate, and medium was changed following the same protocol as for non-immobilized ROs. For live confocal imaging, the rim of the cell culture insert was marked at the 12 and 3 o'clock positions to indicate orientation, and inserts were placed in Cellvis 24-well coverglass-bottom plates. Ubiquitous autofluorescent debris on the PTFE membrane was used as points of reference for the regions of interest. EGFP was excited at 488 nm and collected between 491 nm and 553 nm, mTagRFPT and/or SPY555 were excited at 561 nm and collected between 571 nm and 642 nm, and MitoView 650 was excited at 633 nm and collected between 642 nm and 735 nm. Collected 3D *z*-stack images were processed using Imaris (Version 8.4.2, Oxford Instruments), and individual cone cells were segmented using Imaris Surface function. The inner segment was manually segmented from the cell body at the thinnest connecting point, and the volume was recorded in Imaris. GraphPad Prism was used for statistical analysis with repeated measures one-way ANOVA and to generate graphs.

## Supplementary Material

10.1242/dmm.050193_sup1 Supplementary information

## References

[DMM050193C1] Achberger, K., Probst, C., Haderspeck, J. C., Bolz, S., Rogal, J., Chuchuy, J., Nikolova, M., Cora, V., Antkowiak, L., Haq, W. et al. (2019). Merging organoid and organ-on-a-chip technology to generate complex multi-layer tissue models in a human retina-on-a-chip platform. *ELife* 8, e46188. 10.7554/eLife.46188 31451149 PMC6777939

[DMM050193C2] Aparicio, J., Shayler, D. and Cobrinik, D. (2017). Retinal organoids: an emerging technology for retinal disease research and therapy. In *Cellular Therapies for Retinal Disease* (ed. S. Schwartz, A. Nagiel and R. Lanza), pp. 117-138. Springer. 10.1007/978-3-319-49479-1_10

[DMM050193C3] Aparicio, J. G., Hopp, H., Harutyunyan, N., Stewart, C., Cobrinik, D. and Borchert, M. (2023). Aberrant gene expression yet undiminished retinal ganglion cell genesis in iPSC-derived models of optic nerve hypoplasia. *Ophthalmic Genet*. 10.1080/13816810.2023.2253902 PMC10841193 37807874

[DMM050193C4] Bell, C. M., Zack, D. J. and Berlinicke, C. A. (2020). Human organoids for the study of retinal development and disease. *Annu. Rev. Vis. Sci.* 6, 91-114. 10.1146/annurev-vision-121219-081855 32936736

[DMM050193C5] Browne, A. W., Arnesano, C., Harutyunyan, N., Khuu, T., Martinez, J. C., Pollack, H. A., Koos, D. S., Lee, T. C., Fraser, S. E., Moats, R. A. et al. (2017). Structural and functional characterization of human stem-cell-derived retinal organoids by live imaging. *Invest. Ophthalmol. Vis. Sci.* 58, 3311-3318. 10.1167/iovs.16-20796 28672397 PMC5495152

[DMM050193C6] Capowski, E. E., Samimi, K., Mayerl, S. J., Phillips, M. J., Pinilla, I., Howden, S. E., Saha, J., Jansen, A. D., Edwards, K. L., Jager, L. D. et al. (2019). Reproducibility and staging of 3D human retinal organoids across multiple pluripotent stem cell lines. *Development* 146, dev171686. 10.1242/dev.171686 30567931 PMC6340149

[DMM050193C7] Decembrini, S., Hoehnel, S., Brandenberg, N., Arsenijevic, Y. and Lutolf, M. P. (2020). Hydrogel-based milliwell arrays for standardized and scalable retinal organoid cultures. *Sci. Rep.* 10, 10275. 10.1038/s41598-020-67012-7 32581233 PMC7314858

[DMM050193C8] Gagliardi, G., Ben M'barek, K., Chaffiol, A., Slembrouck-Brec, A., Conart, J. B., Nanteau, C., Rabesandratana, O., Sahel, J. A., Duebel, J., Orieux, G. et al. (2018). Characterization and transplantation of CD73-positive photoreceptors isolated from human iPSC-derived retinal organoids. *Stem Cell Rep.* 11, 665-680. 10.1016/j.stemcr.2018.07.005 PMC6135113 30100409

[DMM050193C9] Gasparini, S. J., Llonch, S., Borsch, O. and Ader, M. (2019). Transplantation of photoreceptors into the degenerative retina: current state and future perspectives. *Prog. Retin. Eye Res.* 69, 1-37. 10.1016/j.preteyeres.2018.11.001 30445193

[DMM050193C10] Gasparini, S. J., Tessmer, K., Reh, M., Wieneke, S., Carido, M., Völkner, M., Borsch, O., Swiersy, A., Zuzic, M., Goureau, O. et al. (2022). Transplanted human cones incorporate into the retina and function in a murine cone degeneration model. *J. Clin. Investig.* 132, e154619. 10.1172/JCI154619 35482419 PMC9197520

[DMM050193C11] Guan, Y., Wang, Y., Zheng, D., Xie, B., Xu, P., Gao, G. and Zhong, X. (2022). Generation of an RCVRN-eGFP reporter hiPSC line by CRISPR/Cas9 to monitor photoreceptor cell development and facilitate the cell enrichment for transplantation. *Front. Cell Dev. Biol.* 10, 870441. 10.3389/fcell.2022.870441 35573687 PMC9096726

[DMM050193C12] Hemshekhar, M., Thushara, R. M., Chandranayaka, S., Sherman, L. S., Kemparaju, K. and Girish, K. S. (2016). Emerging roles of hyaluronic acid bioscaffolds in tissue engineering and regenerative medicine. *Int. J. Biol. Macromol.* 86, 917-928. 10.1016/j.ijbiomac.2016.02.032 26893053

[DMM050193C13] Hendrickson, A. and Zhang, C. (2019). Development of cone photoreceptors and their synapses in the human and monkey fovea. *J. Comp. Neurol.* 527, 38-51. 10.1002/cne.24170 28074469 PMC6529189

[DMM050193C14] Hoshino, A., Ratnapriya, R., Brooks, M. J., Chaitankar, V., Wilken, M. S., Zhang, C., Starostik, M. R., Gieser, L., La Torre, A., Nishio, M. et al. (2017). Molecular anatomy of the developing human retina. *Dev. Cell* 43, 763-779.e4. 10.1016/j.devcel.2017.10.029 29233477 PMC5776731

[DMM050193C15] Kaewkhaw, R., Kaya, K. D., Brooks, M., Homma, K., Zou, J., Chaitankar, V., Rao, M. and Swaroop, A. (2015). Transcriptome dynamics of developing photoreceptors in three-dimensional retina cultures recapitulates temporal sequence of human cone and rod differentiation revealing cell surface markers and gene networks. *Stem Cells* 33, 3504-3518. 10.1002/stem.2122 26235913 PMC4713319

[DMM050193C16] Kelley, M. W., Turner, J. K. and Reh, T. A. (1994). Retinoic acid promotes differentiation of photoreceptors in vitro. *Development* 120, 2091-2102. 10.1242/dev.120.8.2091 7925013

[DMM050193C17] Kuwahara, A., Ozone, C., Nakano, T., Saito, K., Eiraku, M. and Sasai, Y. (2015). Generation of a ciliary margin-like stem cell niche from self-organizing human retinal tissue. *Nat. Commun.* 6, 6286. 10.1038/ncomms7286 25695148

[DMM050193C18] Lam, P. T., Gutierrez, C., Del Rio-Tsonis, K. and Robinson, M. L. (2020). Generation of a retina reporter hiPSC line to label progenitor, ganglion, and photoreceptor cell types. *Transl. Vis. Sci. Technol.* 9, 21. 10.1167/tvst.9.3.21 PMC7352077 32714647

[DMM050193C19] Lu, Y., Shiau, F., Yi, W., Lu, S., Wu, Q., Pearson, J. D., Kallman, A., Zhong, S., Hoang, T., Zuo, Z. et al. (2020). Single-cell analysis of human retina identifies evolutionarily conserved and species-specific mechanisms controlling development. *Dev. Cell* 53, 473-491.e9. 10.1016/j.devcel.2020.04.009 32386599 PMC8015270

[DMM050193C20] Morris, T. A. and Fong, S. L. (1993). Characterization of the gene encoding human cone transducin α-subunit (gnat2). *Genomics* 17, 442-448. 10.1006/geno.1993.1345 8406495

[DMM050193C21] Morris, T. A., Fong, W. B., Ward, M. J., Hu, H. and Fong, S. L. (1997). Localization of upstream silencer elements involved in the expression of cone transducin α-subunit (GNAT2). *Invest. Ophthalmol. Vis. Sci.* 38, 196-206. 9008644

[DMM050193C22] Mustafi, D., Engel, A. H. and Palczewski, K. (2009). Structure of cone photoreceptors. *Prog. Retin. Eye Res.* 28, 289-302. 10.1016/j.preteyeres.2009.05.003 19501669 PMC2740621

[DMM050193C23] Nazlamova, L., Cassidy, E. J., Sowden, J. C., Lotery, A. and Lakowski, J. (2022). Generation of a cone photoreceptor-specific GNGT2 reporter line in human pluripotent stem cells. *Stem Cells* 40, 190-203. 10.1093/stmcls/sxab015 35293574

[DMM050193C24] O'hara-Wright, M. and Gonzalez-Cordero, A. (2020). Retinal organoids: a window into human retinal development. *Development* 147, dev189746. 10.1242/dev.189746 33361444 PMC7774906

[DMM050193C25] Ong, O. C., Hu, K., Rong, H., Lee, R. H. and Fung, B. K. (1997). Gene structure and chromosome localization of the G g c subunit of human cone G-protein (GNGT2). *Genomics* 44, 101-109. 10.1006/geno.1997.4814 9286705

[DMM050193C26] Phillips, M. J., Capowski, E. E., Petersen, A., Jansen, A. D., Barlow, K., Edwards, K. L. and Gamm, D. M. (2018). Generation of a rod-specific NRL reporter line in human pluripotent stem cells. *Sci. Rep.* 8, 2370. 10.1038/s41598-018-20813-3 29402929 PMC5799252

[DMM050193C27] Ran, F. A., Hsu, P. D., Wright, J., Agarwala, V., Scott, D. A. and Zhang, F. (2013). Genome engineering using the CRISPR-Cas9 system. *Nat. Protoc.* 8, 2281-2308. 10.1038/nprot.2013.143 24157548 PMC3969860

[DMM050193C28] Rashidi, H., Leong, Y. C., Venner, K., Pramod, H., Fei, Q. Z., Jones, O. J. R., Moulding, D. and Sowden, J. C. (2022). Generation of 3D retinal tissue from human pluripotent stem cells using a directed small molecule-based serum-free microwell platform. *Sci. Rep.* 12, 6646. 10.1038/s41598-022-10540-1 35459774 PMC9033780

[DMM050193C29] Roberts, B., Haupt, A., Tucker, A., Grancharova, T., Arakaki, J., Fuqua, M. A., Nelson, A., Hookway, C., Ludmann, S. A., Mueller, I. A. et al. (2017). Systematic gene tagging using CRISPR/Cas9 in human stem cells to illuminate cell organization. *Mol. Biol. Cell* 28, 2854-2874. 10.1091/mbc.E17-03-0209 28814507 PMC5638588

[DMM050193C30] Ronning, K. E., Allina, G. P., Miller, E. B., Zawadzki, R. J., Pugh, E. N., Hermann, R. and Goswami, M. (2018). Loss of cone function without degeneration in a novel Gnat2 knock-out mouse. *Exp. Eye Res.* 171, 111-118. 10.1016/j.exer.2018.02.024 29518352 PMC5987249

[DMM050193C31] Seibel, N. M., Eljouni, J., Nalaskowski, M. M. and Hampe, W. (2007). Nuclear localization of enhanced green fluorescent protein homomultimers. *Anal. Biochem.* 368, 95-99. 10.1016/j.ab.2007.05.025 17586454

[DMM050193C32] Shayler, D. W. H., Stachelek, K., Lee, S., Bai, J., Reid, M. W., Aparicio, J. G., Kim, Y., Singh, M. and Cobrinik, D. (2023). Single cell transcriptomics reveals early photoreceptor trajectories and a cancer-predisposed cone precursor state. *BioRxiv*. https://www.biorxiv.org/content/10.1101/2023.02.28.530247v1

[DMM050193C33] Singh, H. P., Wang, S., Stachelek, K., Lee, S., Reid, M. W., Thornton, M. E., Craft, C. M., Grubbs, B. H. and Cobrinik, D. (2018). Developmental stage-specific proliferation and retinoblastoma genesis in RB-deficient human but not mouse cone precursors. *Proc. Natl. Acad. Sci. USA* 115, E9391-E9400. 10.1073/pnas.1808903115 30213853 PMC6176579

[DMM050193C34] Sluch, V. M., Chamling, X., Liu, M. M., Berlinicke, C. A., Cheng, J., Mitchell, K. L., Welsbie, D. S. and Zack, D. J. (2017). Enhanced stem cell differentiation and immunopurification of genome engineered human retinal ganglion cells. *Stem Cells Transl. Med.* 6, 1972-1986. 10.1002/sctm.17-0059 29024560 PMC6430043

[DMM050193C35] Tram, N. K. and Swindle-Reilly, K. E. (2018). Rheological properties and age-related changes of the human vitreous humor. *Front. Bioeng. Biotechnol.* 6, 112-199. 10.3389/fbioe.2018.00199 30619846 PMC6305337

[DMM050193C36] Wahlin, K. J., Maruotti, J. A., Sripathi, S. R., Ball, J., Angueyra, J. M., Kim, C., Grebe, R., Li, W., Jones, B. W. and Zack, D. J. (2017). Photoreceptor outer segment-like structures in long-term 3D retinas from human pluripotent stem cells. *Sci. Rep.* 7, 766. 10.1038/s41598-017-00774-9 28396597 PMC5429674

[DMM050193C37] Wahlin, K. J., Cheng, J., Jurlina, S. L., Jones, M. K., Dash, N. R., Ogata, A., Kibria, N., Ray, S., Eldred, K. C., Kim, C. et al. (2021). CRISPR generated SIX6 and POU4F2 reporters allow identification of brain and optic transcriptional differences in human PSC-derived organoids. *Front. Cell Dev. Biol.* 9, 764725. 10.3389/fcell.2021.764725 34869356 PMC8635054

[DMM050193C38] Wei, X., Henke, V. G., Strübing, C., Brown, E. B. and Clapham, D. E. (2003). Real-time imaging of nuclear permeation by EGFP in single intact cells. *Biophys. J.* 84, 1317-1327. 10.1016/S0006-3495(03)74947-9 12547812 PMC1302708

[DMM050193C39] Welby, E., Lakowski, J., Di Foggia, V., Budinger, D., Gonzalez-Cordero, A., Lun, A. T. L., Epstein, M., Patel, A., Cuevas, E., Kruczek, K. et al. (2017). Isolation and comparative transcriptome analysis of human fetal and iPSC-derived cone photoreceptor cells. *Stem Cell Rep.* 9, 1898-1915. 10.1016/j.stemcr.2017.10.018 PMC5785701 29153988

[DMM050193C40] Xu, X. L., Singh, H. P., Wang, L., Qi, D. L., Poulos, B. K., Abramson, D. H., Jhanwar, S. C. and Cobrinik, D. (2014). Rb suppresses human cone-precursor-derived retinoblastoma tumours. *Nature* 514, 385-388. 10.1038/nature13813 25252974 PMC4232224

[DMM050193C41] Zhong, X., Gutierrez, C., Xue, T., Hampton, C., Vergara, M. N., Cao, L. H., Peters, A., Park, T. S., Zambidis, E. T., Meyer, J. S. et al. (2014). Generation of three-dimensional retinal tissue with functional photoreceptors from human iPSCs. *Nat. Commun.* 5, 4047. 10.1038/ncomms5047 24915161 PMC4370190

