# Supplementary material for: Episodic live imaging of cone photoreceptor maturation in GNAT2-EGFP retinal organoids
Source: Dis Model Mech. 2023 Nov 21;16(11):dmm050193. doi: 10.1242/dmm.050193 (PMC10690052; doi:10.1242/dmm.050193)
Supplement: Supplementary information [file dmm-16-050193-s1.pdf]

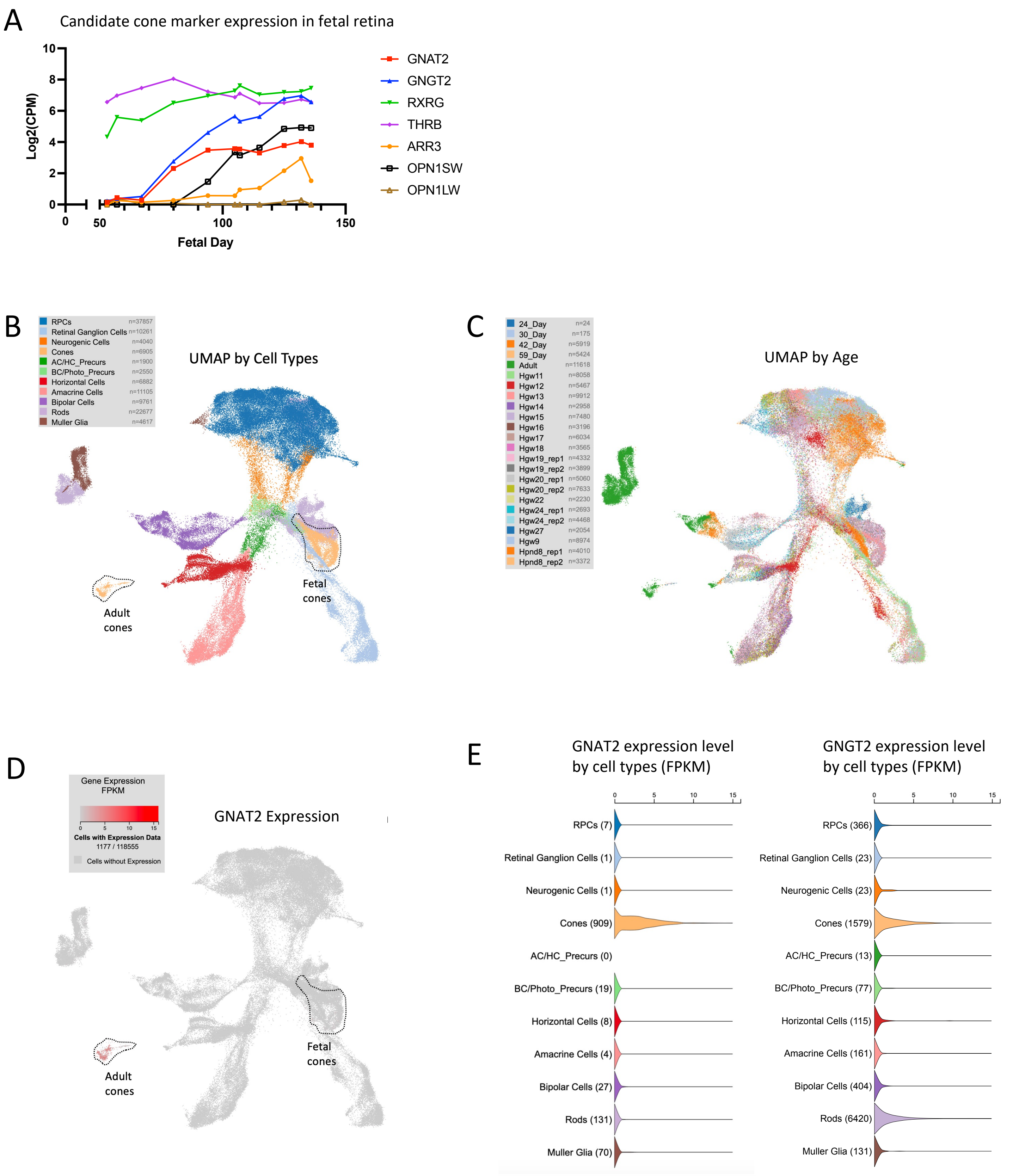

Disease Models & Mechanisms • Supplementary information

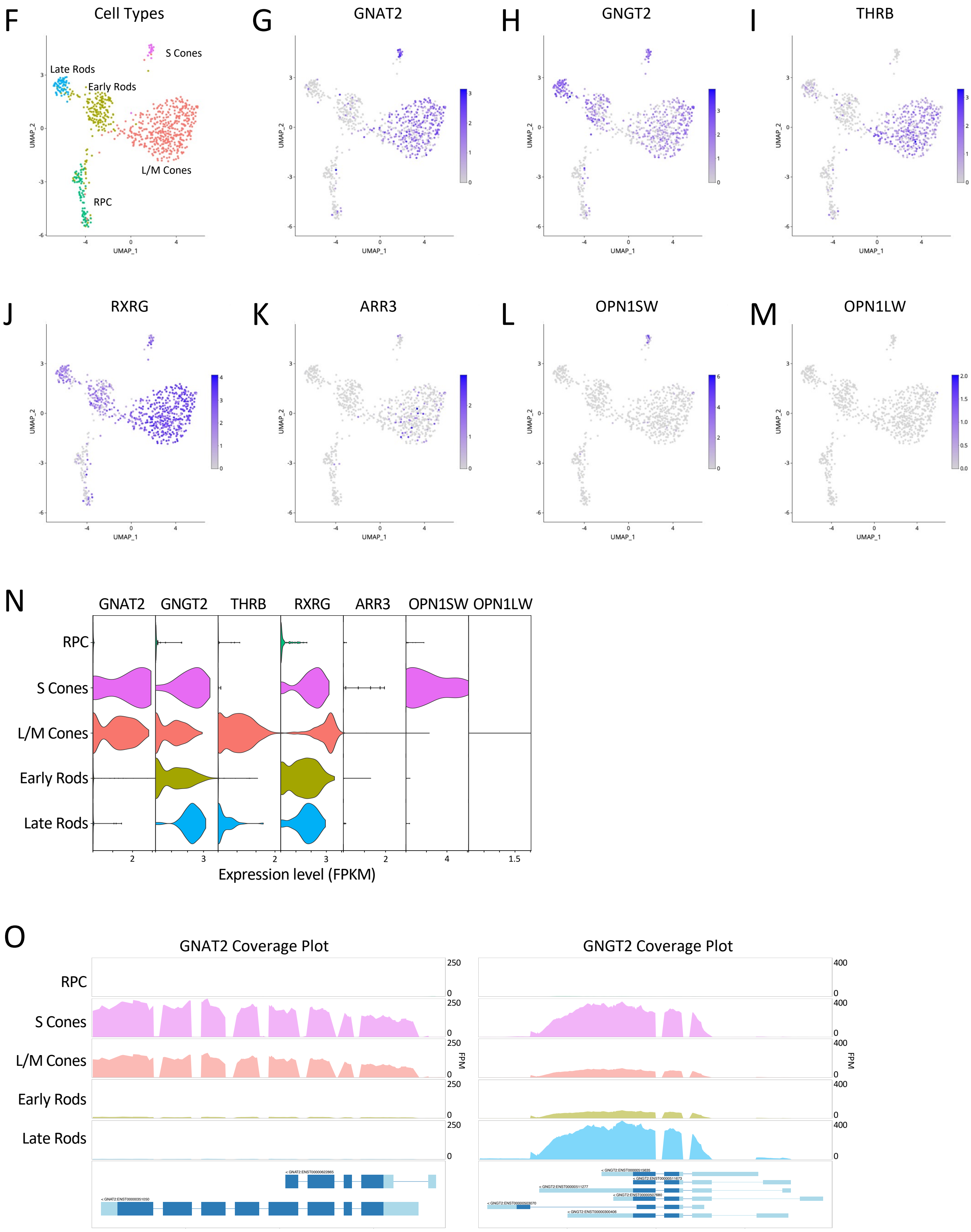
F
Cell Types
S Cones
Late Rods
Early Rods
L/M Cones
RPC
UMAP_1
UMAP_2
G
GNAT2
H
GNGT2
I
THRB
J
RXRG
K
ARR3
L
OPN1SW
M
OPN1LW
N
GNAT2 GNGT2 THRB RXRG ARR3 OPN1SW OPN1LW
RPC
S Cones
L/M Cones
Early Rods
Late Rods
Expression level (FPKM)
O
GNAT2 Coverage Plot
GNGT2 Coverage Plot
RPC
S Cones
L/M Cones
Early Rods
Late Rods
FPM

Fig. S1. Cone-specific *GNAT2* expression in scRNA-seq analyses.

(A) Expression of seven candidate cone markers in developing fetal retina. Bulk RNA-seq data (GSE104827) from Hoshino et al., 2017. (B-D) 3D UMAP display of scRNA sequencing of early stage ROs, fetal retinae, and adult retinae from Lu et al., 2020, by cell types (B), tissue age (C) and *GNAT2* expression (D). (E) Violin plot of *GNAT2* and *GNGT2* expression by cell type from the same scRNA sequencing dataset. (F-M) 2D UMAP display of scRNA sequencing (GSE207802) of FACS-enriched fetal retinal progenitor cells (RPCs) and photoreceptors from (Shayler et al., 2023) by cell type (F) and by expression of cone markers *GNAT2* (G), *GNGT2* (H), *THRB* (I), *RXRG* (J), *ARR3* (K), *OPN1SW* (L), and *OPN1LW* (M). (N) Violin plots of cone marker expression by cell type in the same full-length scRNA sequencing dataset. (O) Exon coverage plot of *GNAT2* and *GNGT2* reads by cell type in the same full-length scRNA sequencing. ENSEMBL transcript isoforms are shown below each plot. No cell type-specific isoform usage was detected.

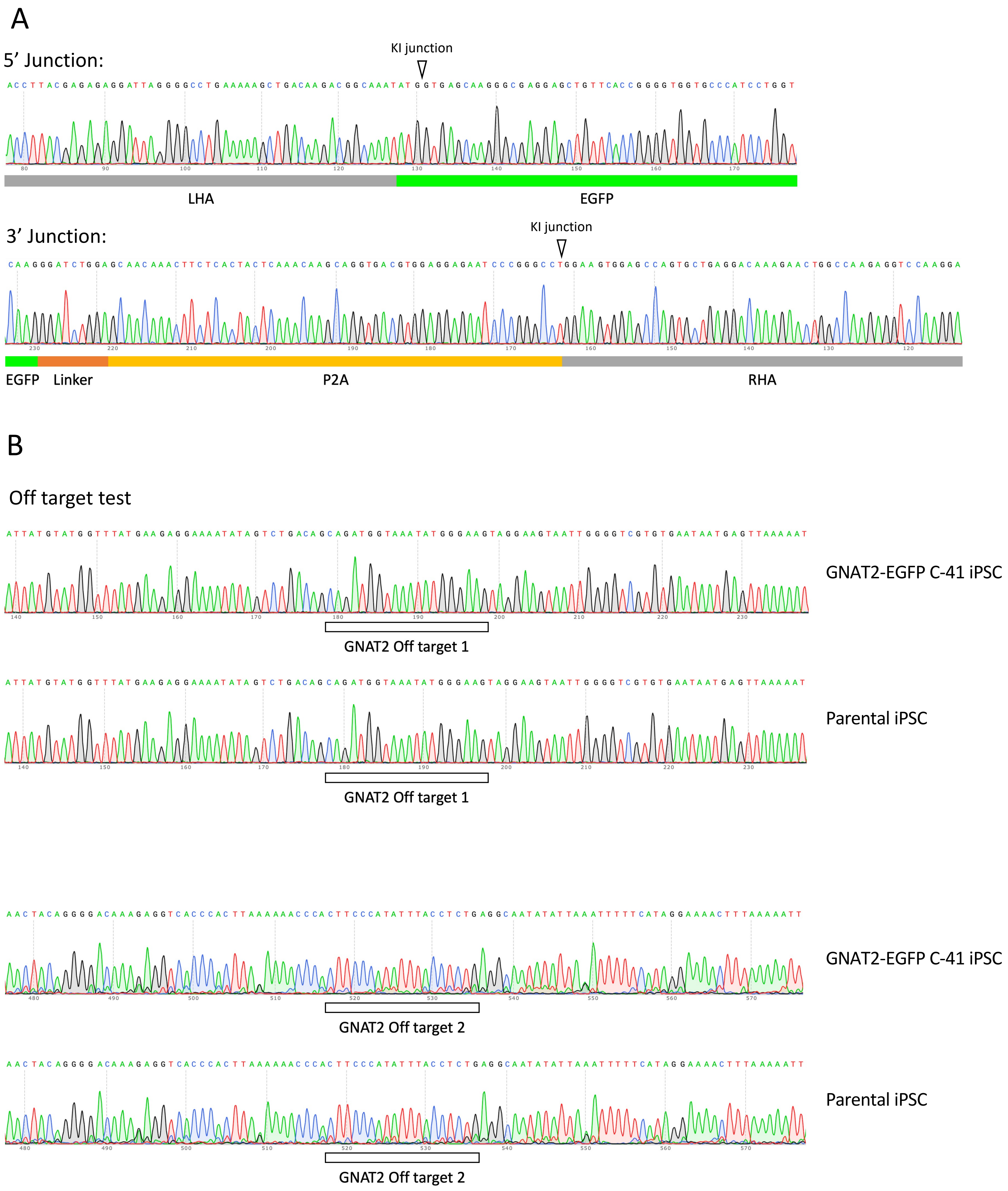

Disease Models & Mechanisms • Supplementary information

B cont.

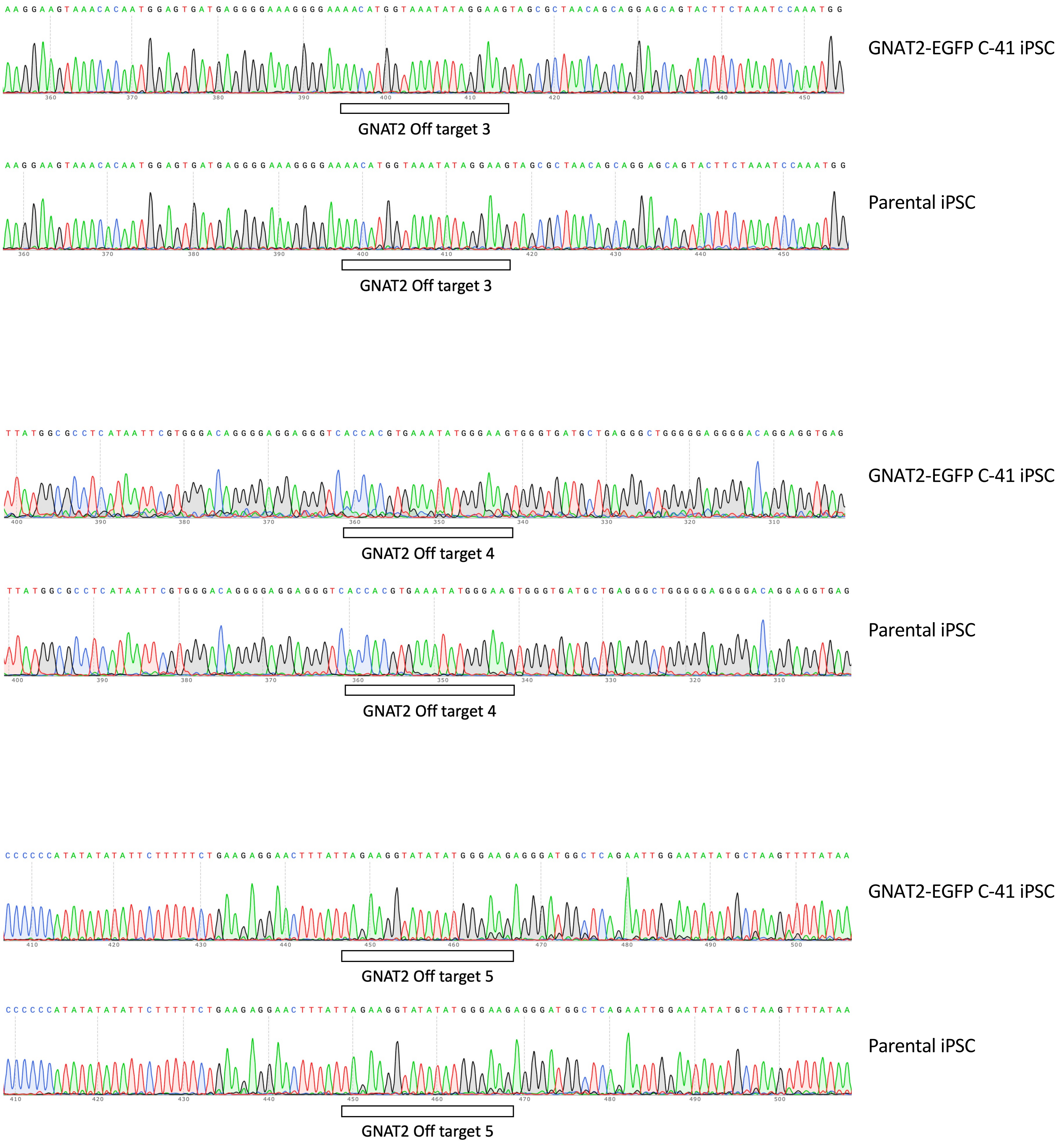

Disease Models & Mechanisms • Supplementary information

C

GNAT2-GFP C-41 Karyotype

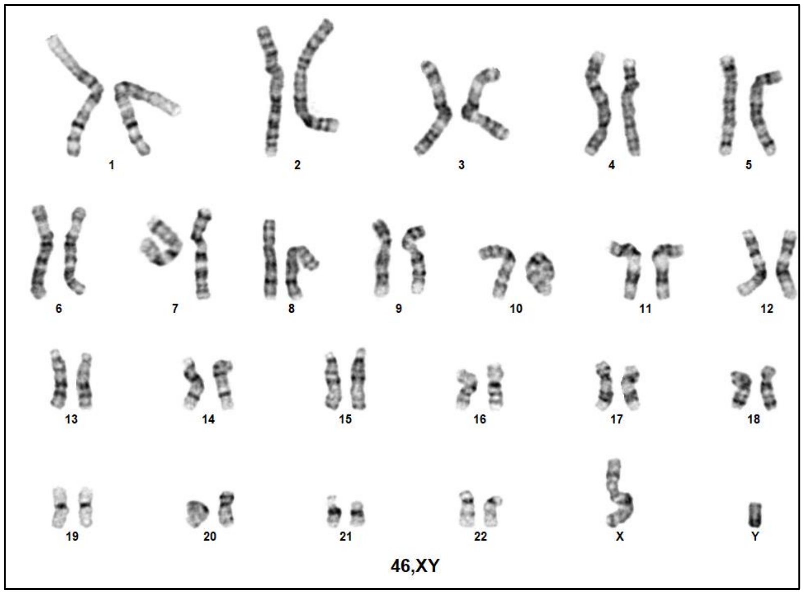

Fig. S2. GNAT2-EGFP C-41 genotyping, off-target sequencing and karyotype.

(A) Sanger sequencing of the 5' end and 3' knock-in junctions showing expected junction sequences. (B) Sanger sequencing showing no mutations detected at the top five predicted off-target sites of the gRNA used for CRISPR knock-in. (C) GNAT2-EGFP C-41 shows normal karyotype.

Disease Models & Mechanisms • Supplementary information

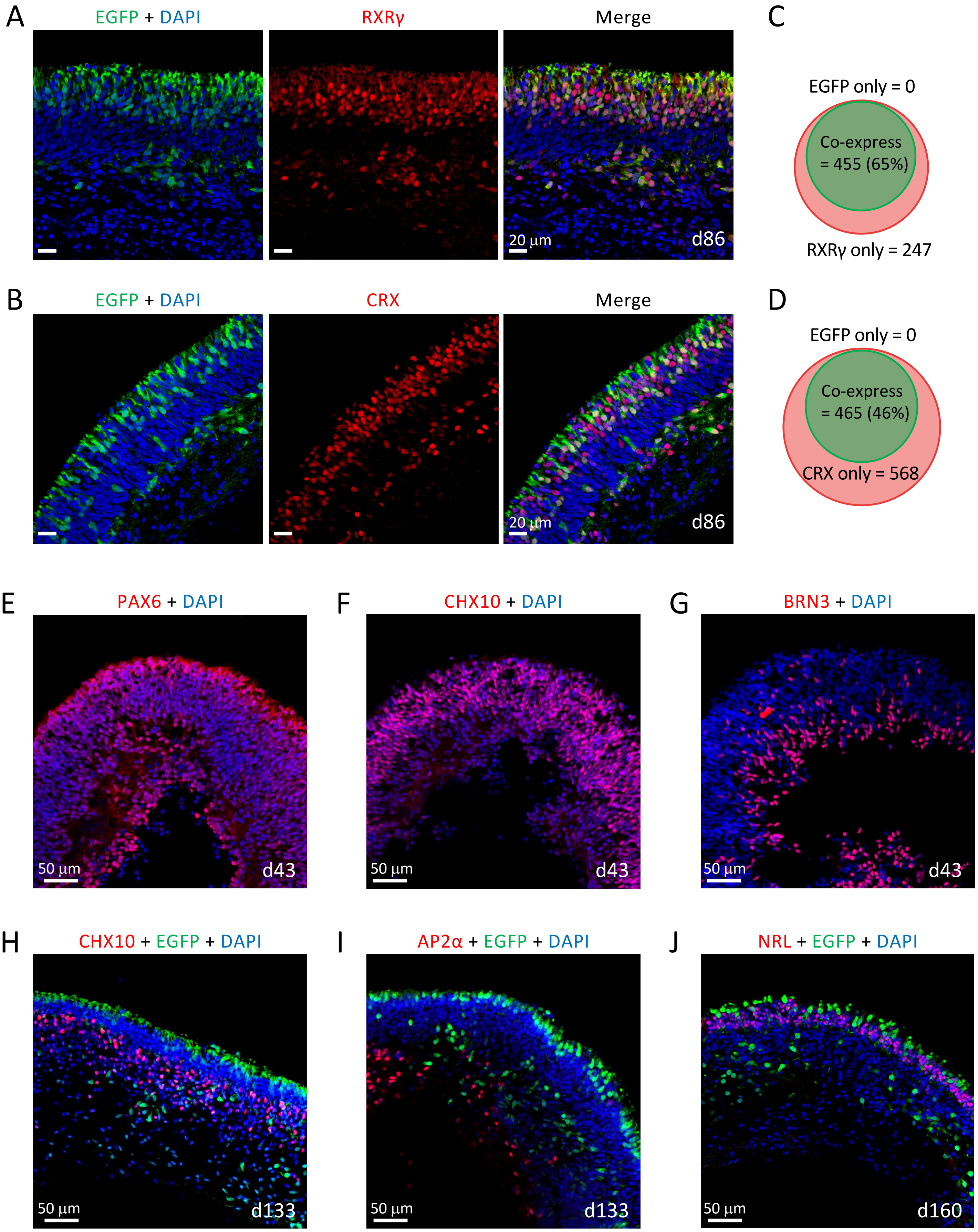

Disease Models & Mechanisms • Supplementary information

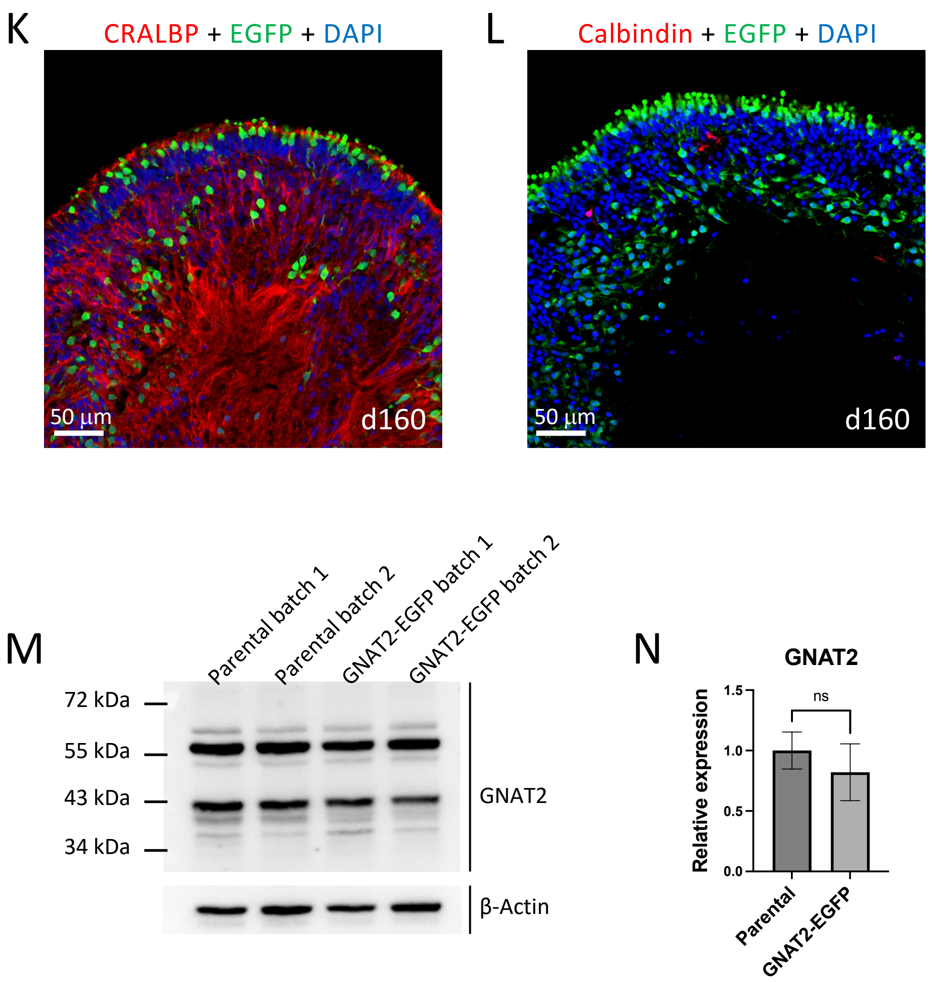

Fig. S3. GNAT2-EGFP RO characterization.

(A, B) Representative immunostaining of d86 GNAT2-EGFP ROs indicating co-expression of EGFP and RXRγ (A) or EGFP and CRX (B). Scale bars: 20 µm. (C) Quantification of cells expressing EGFP only, RXRγ only, or both in four sections from three ROs. (D) Quantification of cells expressing EGFP only, CRX only, or both in four sections from three ROs. (E-L) Immunostaining of GNAT2-EGFP ROs indicating the presence of retinal progenitor cells (PAX6 and CHX10) at d43 (E, F), retinal ganglion cells (BRN3) at d43 (G), bipolar cells (CHX10) at d133 (H), amacrine cells (AP2α) at d133 (I), rod photoreceptors (NRL) at d160 (J), Müller glia (CRALBP) at d160 (K), and horizontal cells (Calbindin) at d160 (L). Scale bars: 50 µ m. (M) GNAT2 western blot in d70 ROs differentiated from the parental iPSC line or the edited GNAT2-EGFP C-41. This GNAT2 antibody detects a band at the expected size of 41 kDa a nd a band of unknown significance at 57 kDa. Each lane is from an independent batch of RO differentiation. (N) Quantification of GNAT2:β-Actin ratios in (M). Error bar = SD. Two-tailed t-test gave p=0.462.

Disease Models & Mechanisms • Supplementary information

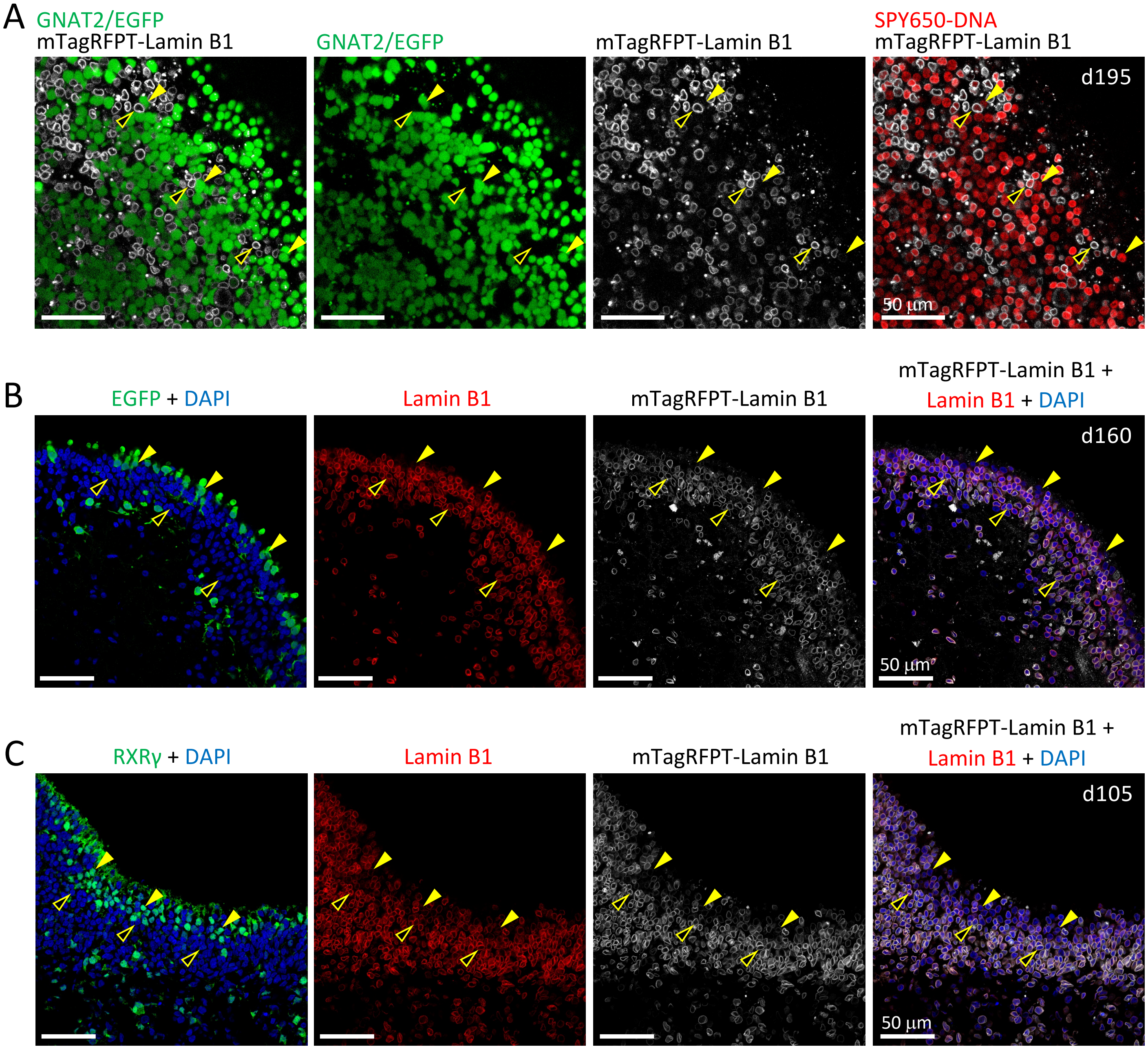

Disease Models & Mechanisms • Supplementary information

Fig. S4. Decreased mTagRFPT-Lamin B1 expression in EGFP+ cones.
(A) Single Z-section from a Z-stack live confocal image of a GNAT2-EGFP RO at d195 showing endogenous EGFP and mTagRFPT. Nuclei are labeled with SPY650 DNA dye. Solid yellow arrowheads indicate low mTagRFPT-Lamin B1 expression in EGFP+ cones, while empty yellow arrowheads indicate high mTagRFPT-Lamin B1 expression in EGFP-negative non-cone retinal cells. Scale bars: 50 μm. (B) Immunostaining of d160 GNAT2-EGFP RO sections showing decreased Lamin B1 protein and mTagRFPT-Lamin B1 signal in EGFP+ cones (solid yellow arrowheads) compared to EGFP-negative non-cone retinal cells (empty yellow arrowheads). Scale bars: 50 μm. (C) Immunostaining of d105 WTC-mTagRFPT-LMNB1 RO sections showing decreased Lamin B1 protein and mTagRFPT-Lamin B1 signal in RXRγ+ cones (solid yellow arrowheads) compared to RXRγ-negative non-cone retinal cells (empty yellow arrowheads). Scale bars: 50 μm.

Table S1. Top five off-targets predicted by IDT CRISPR-Cas9 gRNA checker.

Sequence (5'-3')	PAM	Score	#MM	Gene	Locus
AAGACGGCAAATATGGGAAG	TGG	N/A		GNAT2	chr1:-109612862
CAGATGGTAAATATGGGAAG	TAG	0	3		chr7:+15609261
CAGA-GGTAAATATGGGAAG	TGG	3	3		chr10:-107997752
AACATGGTAAATATAGGAAG	TAG	8	4		chr10:+26802625
ACCACGTGAAATATGGGAAG	TGG	9	4		chr22:-26429847
TAGAAGGTATATATGGGAAG	AGG	9	4		chr4:-61246789

Disease Models & Mechanisms • Supplementary information

Table S2. Oligonucleotide primers (5'-3') used in this study.

**gRNA sequence**	
GNAT2 gRNA	AAGACGGCAAATATGGGAAG
**Cloning primers**	
GNAT2 sgRNA F	CACCGAAGACGGCAAATATGGGAAG
GNAT2 sgRNA R	AAACCTTCCCATATTTGCCGTCTTC
pUC118 F	GAACATTTGAGACACGTGGCTTAAGACCTGCAGGCATGCAAG
pUC118 R	GGTGTATGGTGAAGAGAATGGGGACTCTAGAGGATCCCCGGG
EGFP F	AAGACGGCAAATATGGTGAGCAAGGGCGAGGAG
EGFP R	ACTGGCTCCACTTCCAGGCCCGGGATTCTCCTC
GNAT2 LHA F	CCCATTCTCTTCACCATACACCCT
GNAT2 LHA R	ATTTGCCGTCTTGTCAGCTTTTTC
GNAT2 RHA F	GGAAGTGGAGCCAGTGCTGA
GNAT2 RHA R	AGCCACGTGTCTCAAATGTTCTTG
**Primers for GNAT2-EGFP genotyping**	
Location-specific F	GCTGGCTTTAGTGGGCCAAA
Location-specific R	CTTGTGGCCGTTTACGTCGC
Insert-flanking F	TACAGCCTGCTCTCTCACCT
Insert-flanking R	AGGTAGAACCCACCAACCCT
**Primers for amplifying GNAT2-EGFP off target regions**	
Off-target 1F	GCCTCTGATCTTAGTTTACTAGTCT
Off-target 1R	ACTGACAAACAGGGAAGCCA
Off-target 2F	TCCCAACCATAGCACTTGGC
Off-target 2R	AGTTTGCTTTTGCCCAGCAT
Off-target 3F	ATTAAGGCAGGCGCCAGATG
Off-target 3R	GGAAGCAGTGAGAAAGGCCA
Off-target 4F	GTGGGAGTTGTGGTCTTCGC
Off-target 4R	AAGGAGGTTTATGGCGCCTC
Off-target 5F	ATGAGCTAGATGGTGCCTGA
Off-target 5R	AACAGAATGCAATGAAGGCTCT

Table S3. Primary antibodies used in this study.

Antibody	Supplier company	Catalog #	Dilution
Goat anti-GFP	Abcam	ab6673	1:400
Rabbit anti-GFP	Thermo Fisher Scientific	A11122	1:200
Mouse anti-ARR3	Millipore Sigma	MABN2636	1:500
Rabbit anti-RXRγ	Santa Cruz Biotechnology	sc-555	1:1000
Rabbit anti-TOM20	Proteintech	11802-1-AP	1:200
Mouse anti-CRX	Abnova	H00001406-M02	1:1000
Rabbit anti-PAX6	Biolegend	90130	1:50
Sheep anti-CHX10	ExAlpha Biologicals	X1179P	1:250
Goat anti-BRN3	Santa Cruz Biotechnology	sc-6026	1:50
Mouse anti-NRL	Santa Cruz Biotechnology	sc-374277	1:50
Mouse anti-AP2α	Developmental Studies Hybridoma Bank	DSHA-3B5	1:18
Mouse anti-CRALBP	Abcam	ab15051	1:250
Rabbit anti-Calbindin	Abcam	ab1778	1:500
Mouse anti-Lamin B1	Biolegend	869801	1:500
Rabbit anti-GNAT2	Thermo Fisher Scientific	PA5-22340	1:1000
Rabbit anti-β-Actin	Cell Signaling Technology	4967S	1:1000
